# A comparative study of freeze-drying heat transfer in polymeric vials and glass vials

**DOI:** 10.1038/s41598-023-40777-3

**Published:** 2023-10-23

**Authors:** Morteza Sarmadi, Spencer Holmes, Royal Agha, Brandon Davenport, Christopher Weikart, T. N. Thompson

**Affiliations:** 1 SiO2 Materials Science, Auburn, AL 36830 USA; 2 Millrock Technology, Kingston, NY 12401 USA

**Keywords:** Mechanical engineering, Biomaterials, Biomedical materials, Energy science and technology, Engineering, Materials science

## Abstract

Implementation of polymeric vials for freeze-dried drug products has been practically non-existent because of unique moisture barrier and thermodynamic technical challenges. Hybrid vials, which combine the benefits of polymer and glass, have been shown to address the challenges of ordinary polymeric vials. Tackling thermodynamic challenges starts with a clear understanding of the heat transfer mechanism. To this end, multi-physics simulations and experimentation were used to compare the heat transfer between hybrid cyclic olefin polymer (COP) vials and borosilicate glass vials during freeze-drying. Parametric models were developed for hybrid COP and glass vials to systematically study the effect of five design parameters based on the arrangement of the vials on a tray inside a lyophilization chamber. Heat transfer in glass vials were dominated by heat conduction with the surrounding vapor, while hybrid COP vials were governed by conduction with the bottom shelf. Furthermore, hybrid COP vials exhibited more consistent heat flow rate and total heat transfer coefficient compared to glass vials, suggesting higher product quality as a result. The distance between adjacent vials and the drug product height were the most important parameters affecting heat transfer irrespective of vial type. Results indicated that hybrid COP vials can be filled to higher fill volumes with higher heat transfer and without the risk of breakage. Results of this study can help design innovative primary packaging systems for freeze drying or optimizing heat transfer for existing glass or hybrid COP vial systems regarding product consistency and drying time.

## Introduction

Polymeric vials provide benefits over ordinary glass vials such as breakage resistance, dimensional control, and design flexibility. The pharmaceutical packaging industry has been trying to exploit these benefits for liquid injectable biologic drug products for many years. Polymeric vials, however, have inferior gas and moisture barrier properties that are deleterious for the stability and shelf-life for both liquid and freeze-dried biologic drugs. A new hybrid vial was developed by SiO_2_ Materials Science (SiO_2_) that combined the benefits of glass and plastic, but without their respective drawbacks. SiO_2_ has a proven track record of commercial success for liquid injectable biologic drugs and vaccines. The unique synergy of glass and plastic in SiO_2_ vials solves both thermodynamic and barrier property shortcomings that cannot be solved by plastic alone^1–3^. Both properties are key to a successful vial for freeze-dried drug product. The barrier properties of SiO_2_ vials are beyond the scope of this study and have been showcased in prior publications^1–3^. The thermodynamic properties of SiO_2_ vials for freeze-dried drugs is the focus of this paper.

Freeze-drying or lyophilization is a widely-utilized process to preserve a broad range of sensitive biopharmaceutical drugs, micro-organisms, nanoparticles, and food products^1–5^. The expected global market size for freeze-drying by the end of 2022 is predicted to be US$ 1.1 billion dollars growing at a compound annual growth rate (CAGR) of 8.5% to reach a total market size of around US$ 2.5 billion by the year 2032^4^. A key application of freeze-drying is advanced biotechnology drug and vaccine products to extend their shelf-life stability and more convenient shipping^5,6^. Freeze-drying has been a well-established technology for drug products for decades and expected to experience continued growth reaching approximately $4 billion, growing at a CAGR of 5.8% from 2022 to 2028^7^. Lyophilization holds significant promises for extending the half-life for temperature sensitive biologics and vaccines. This is an attractive alternative to ordinary frozen storage, which involves complicated and costly cold chain distribution issues as suggested by some studies^6,8^.

Freeze-drying consists of three major steps, namely, freezing, primary drying and secondary drying. Briefly, during the freezing step, the temperature of the aqueous solution drops below its triple point whereby liquid water, solid ice and water vapor coexist at equilibrium. In primary drying, the pressure drops while maintaining the freezing temperature to allow the solid ice phase to sublimate to water vapor. Finally, during the secondary drying, sublimation continues by increasing temperature and maintaining low pressures (e. g. < 120 mTorr) to remove any remaining unfrozen or sorbed water. A full description of the freeze-drying process steps can be found in the literature^9,10^. The thermodynamics of freeze-drying drug products inside primary containers is complex and dependent on multiple factors such as freezing rate, water crystallization, chamber pressure, shelf temperature, rate of drying, position of the sample and so forth^9–11^.

In the biopharmaceutical industry, drug formulations are freeze-dried inside of primary packaging such as vials. The design of vials and their arrangements within the freeze-drying chamber has been shown to play an important factor in heat transfer during freeze-drying, therefore, the quality and the time spent for achieving consistent products^12–14^. Vials made from Type I borosilicate glass have been commonly used for storage of pharmaceutical products during freeze-drying. However, the risk of breakage, lack of chemical compatibility (i.e., formulation pH), low fill-volume, lack of design flexibility, and low manufacturing throughput has propelled innovations towards development of new classes of vials, especially for sensitive and expensive biologic drugs^14–16^. A new generation of vials made from polymers has accordingly gained attention in recent years^14,17,18^. Vials made from polymers such as cyclic olefin polymer (COP) and cyclic olefin copolymer (COC) have been shown to solve some, but not all of the problems of traditional glass vials^14,17–20^. Particularly for freeze-drying, polymer vials are excessively permeable to water vapor, which can compromise the ability to keep the drug product dry during storage. More recently, hybrid COP vials were developed to combine the benefits of polymeric and glass materials, as fully described in Refs.^1–3^. Hybrid polymeric structure corresponds to having a hybrid combination of a polymeric vial body with a microscopic layer of SiO_2_ coated on the inner surface of the vial. The plasma-enhanced chemical vapor deposition (PECVD) is implemented to apply the coating on the inner surface of the vials, forming a barrier at the interface between the vial bulk material and the product solution within the vial. The high density and inert chemical make-up of the layer limits water vapor entry and harmful interactions with the drug product^1–3^. Despite the promising features of hybrid COP vials, there is no study to date that has systematically investigated heat transfer during freeze-drying in polymeric vials compared with glass vials.

Understanding heat transfer during various steps of freeze-drying of the vials containing the product is critical to having high quality products with consistent physicochemical properties^8–10^. While being an essential step in preparation of pharmaceutical products, freeze-drying is a costly and time-consuming operation^10,15^. The ability to predict and model heat transfer in vials can be highly beneficial to reduce sample-to-sample variability and processing time, and to increase throughput. Computational modeling of freeze-drying has been shown to be a reliable tool for optimizing heat transfer during freeze-drying, and widely utilized in the pharmaceutical industry^12,20,21^. Due to the underlying complexity of the freeze-drying process and geometrical design of the vials, modeling heat transfer by traditional mathematical tools can be extremely difficult. This is especially the case when considering interactions caused by radiation between various components (e.g., chamber wall, adjacent vials, product, etc.) interacting inside a freeze-drying chamber^12,22,23^. Advanced computational tools such as multi-physics finite element analysis can be highly useful in this regard. Recently, integration of multi-physics modeling with powerful statistical tools such as design of experiment (DoE) and analysis of variance (ANOVA) has been demonstrated to be highly effective in providing a systemic framework for design and optimization of medical devices^24,25^.

In this study, we use advanced multi-physics simulations to study heat transfer during primary drying in polymeric hybrid COP vials and glass vials. To this end, we consider a new generation of vials made from hybrid COP with a flat bottom, as a model for polymeric vials. Prior studies have shown that the heat transfer characteristics of COP vials with or without the microscopic coating are similar^1–3^. Multi-physics simulations are then conducted for a representative finite element model of 13 vials in a freeze-drying tray, independently for hybrid COP and glass vials at identical boundary conditions. Once experimentally validated, the simulation models are utilized to study contributions stemming from major modes of heat transfer. The modes include (1) conduction from the vapor surrounding the vial, (2) conduction from the bottom shelf in contact with the vials, and (3) radiation from various freeze-drying components. Next, we generate a DoE table and study the effect of five design parameters, commonly used in industrial scale freeze-drying equipment, on the heat transfer behavior in hybrid COP and glass. Collectively, more than 100 simulations are carried out to construct a comprehensive framework to compare heat transfer between hybrid COP and glass vials during primary-drying. These simulations can be used to guide design optimizations in vials regarding material properties, vial geometry, and vial arrangement for industrial scale freeze-drying applications.

## Results

**Figure 1:**
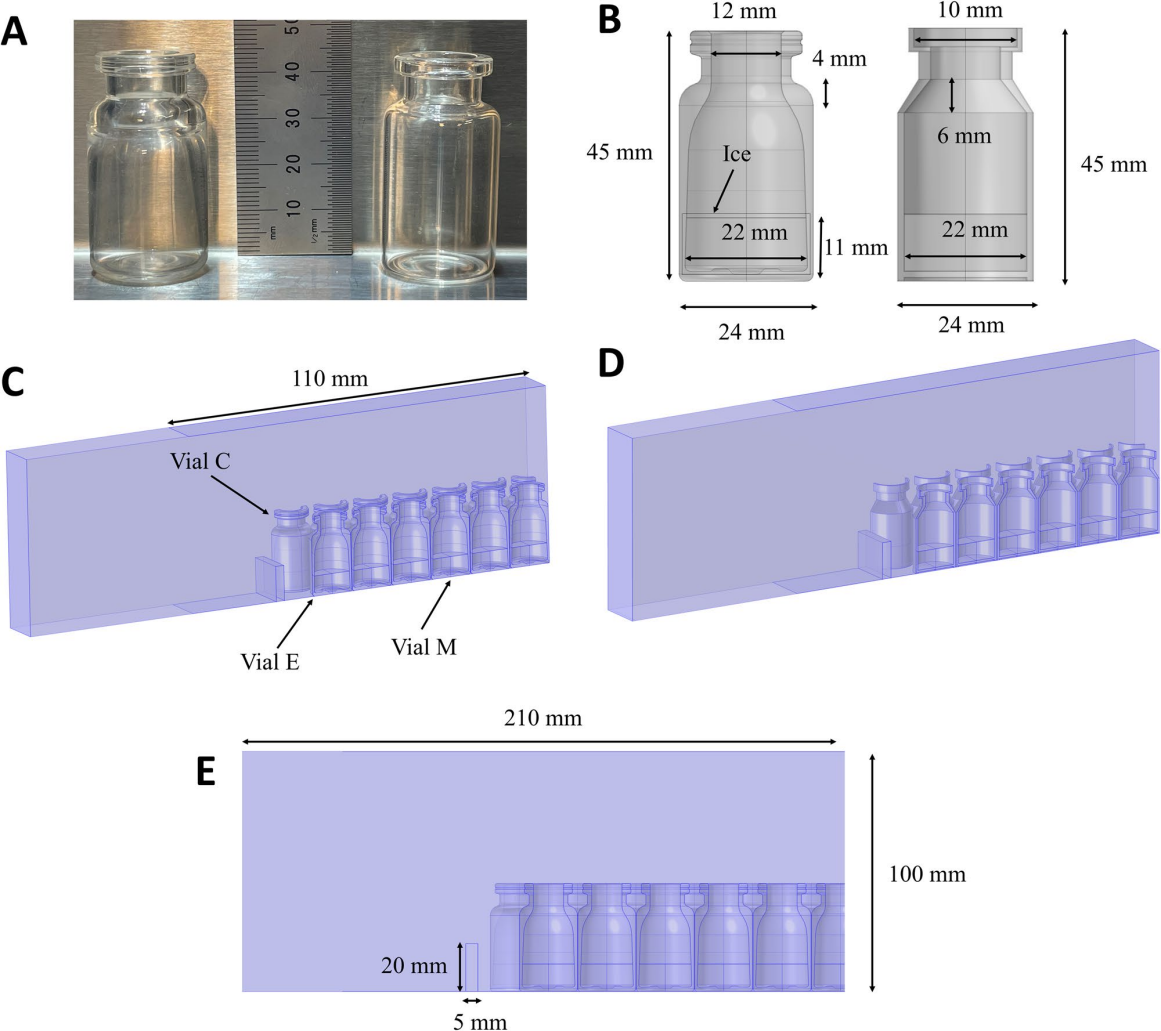
Dimensions and spatial arrangement of hybrid COP and glass vials investigated in this study. (**A**) Actual picture of an individual hybrid COP vial (left) and a glass vial (right)—both at 10 mL nominal volume. (**B**) Simplified models of an individual hybrid COP vial (left) and a glass vial (right) containing ice as the model freeze-drying product. (**C**) Depiction of the full multi-physics model special arrangement of 13 vials sitting on a freeze-drying shelf for (**C**) hybrid COP and (**D**) glass vials showing three different types of vials studied (i.e., Vial C, Vial E and Vial M). (**E**) Side view of the full model used for hybrid COP vials. All spatial dimensions are the same in both vial models, but the hybrid COP and glass vials themselves have slightly different dimensions.

To compare the heat transfer behavior during freeze-drying between hybrid COP and glass vials, we considered vials with similar dimensions but made from different materials. The glass vial was composed of type 1 borosilicate glass (Schott 10R) and compared with a hybrid COP vial (SiO_2_ Materials Science), both with a nominal volume of 10 mL. A side-by-side picture of the two vials is presented in Fig. 1A. Further, Fig. 1B shows a simplified model of each vial type used for multi-physics simulations. Next, an expanded model of the vials was generated for each vial type as a representative volume element (RVE) (Fig. 1C,D). The expanded model and nomenclature used to label relative positions of the vials were inspired by the work of Scutella et al.^12^ which has shown very good agreement with experimental results. In the RVE models, half or a quarter of vials were incorporated accounting for 13 rows of vials next to each other. Thermal properties of materials used in this study can be found in Table S1 and section “Materials and methods”. Vials located at three different positions were studied separately, namely (1) vials in contact with or closest to the freeze-drying rail-guard or “rail” (vial C), (2) vials next to the rail but not in contact with the rail (vial E), and (3) vials forming bulk of the vials (vial M or central vial) located far away from the rail by 5 rows (Fig. 1C). An expanded model was used with identical boundary conditions for both hybrid COP and glass vial type I (Fig. 1C,D).

**Figure 2:**
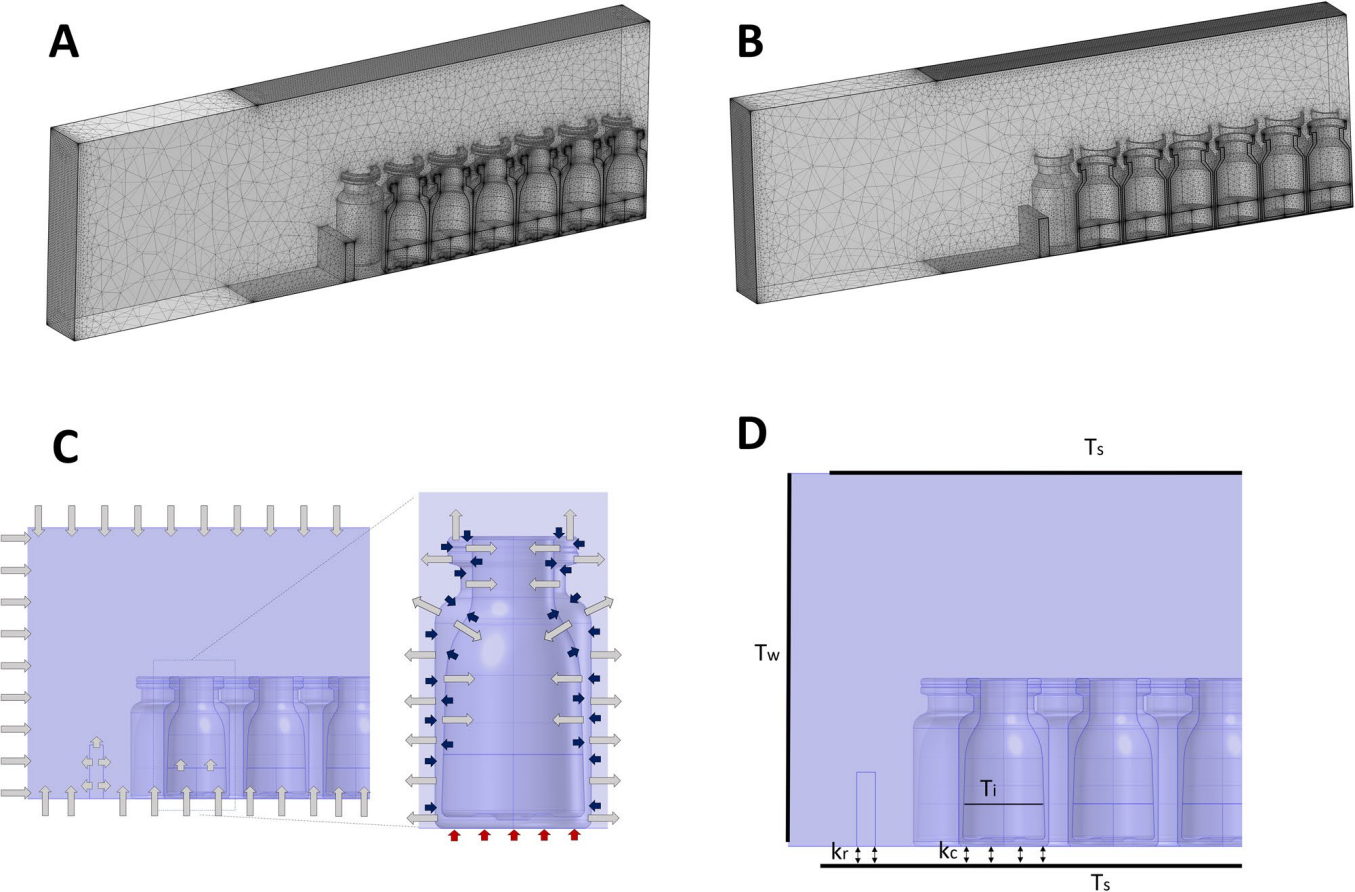
Details of the multiphysics models used in the simulations. Overview of the physically optimized meshed model of (**A**) hybrid COP vials and (**B**) glass vials using a “finer” mesh setting in COMSOL. (**C**) Schematic illustration of modes of heat transfer defined in the Multiphysics simulations. Gray arrows show radiation from the surface of solid objects (i.e., wall, ice, rail, shelves, vials), blue arrow shows contact conduction from the vapor surrounding the vials, and red arrow shows contact conduction from bottom shelf to the bottom surface of the vial (vial-shelf interface). Note that each vial both emits radiation to and receives radiation from surrounding vials. The gray arrows shown on an individual vial only depict emission of radiative heat flux for sake of simplicity. (**D**) Overview of the boundary conditions (i.e., temperatures and contact conduction heat transfer coefficients) used to define the model.

The same arrangement of vials shown in the model (Fig. 1) was implemented in experiments (Fig. 2). The total heat transfer coefficient of vial (K_v_) was measured for each vial type at a shelf temperature (T_s_) of 0 °C or – 20 °C and freeze-drying chamber pressures (P_c_) of 30, 68, and 112 mTorr. The resulting values (n = 3 runs, 322 vials used in each run) are represented individually for each type of vial regarding various vial positioning (vials C, E, and M). The K_v_ increased for vials located closer to the rail—going from vial M to E, and C—due to the edge vial effect. The measured K_v_ for hybrid COP was relatively lower than that of glass at T_s_ = 0 °C (e.g., up to 8–19% lower across all chamber pressures in vial M). But at T_s_ = − 20 °C, K_v_ for hybrid COP was statistically similar to glass (p-value > 0.05 in all three vial positions). Hybrid COP vials represented a lower standard deviation of K_v_ compared to glass vials. Standard deviation of K_v_ for hybrid COP vials compared to glass vials was lower by 10%, 8%, 12%, corresponding to vial C, E, and M, respectively (averaged over all experimental conditions). The difference between average K_v_ of vial C relative to vial M, representing deviation in the heat flow rate in edge vs central vials, was lower for hybrid COP vials in most cases. Accordingly, at T_s_ = 0 °C, this parameter for glass vials was greater than that of hybrid COP vial by 34, 0, and 24%, at P_c_ = 30, 68, and 112 mTorr, respectively. These results collectively suggest higher product consistency among hybrid COP vials compared to glass, despite lower K_v_ values, matching well with results from a similar study on hybrid vials^3^. Product consistency refers to the uniformity or lack of variation in the heat transfer coefficient between edge vials and center vials, captured by a lower percentage of difference of K_v_ between edge VS center vials. In other words, it indicates the degree to which there are no significant differences in the heat transfer performance across various vials in the same batch.

**Figure 3:**
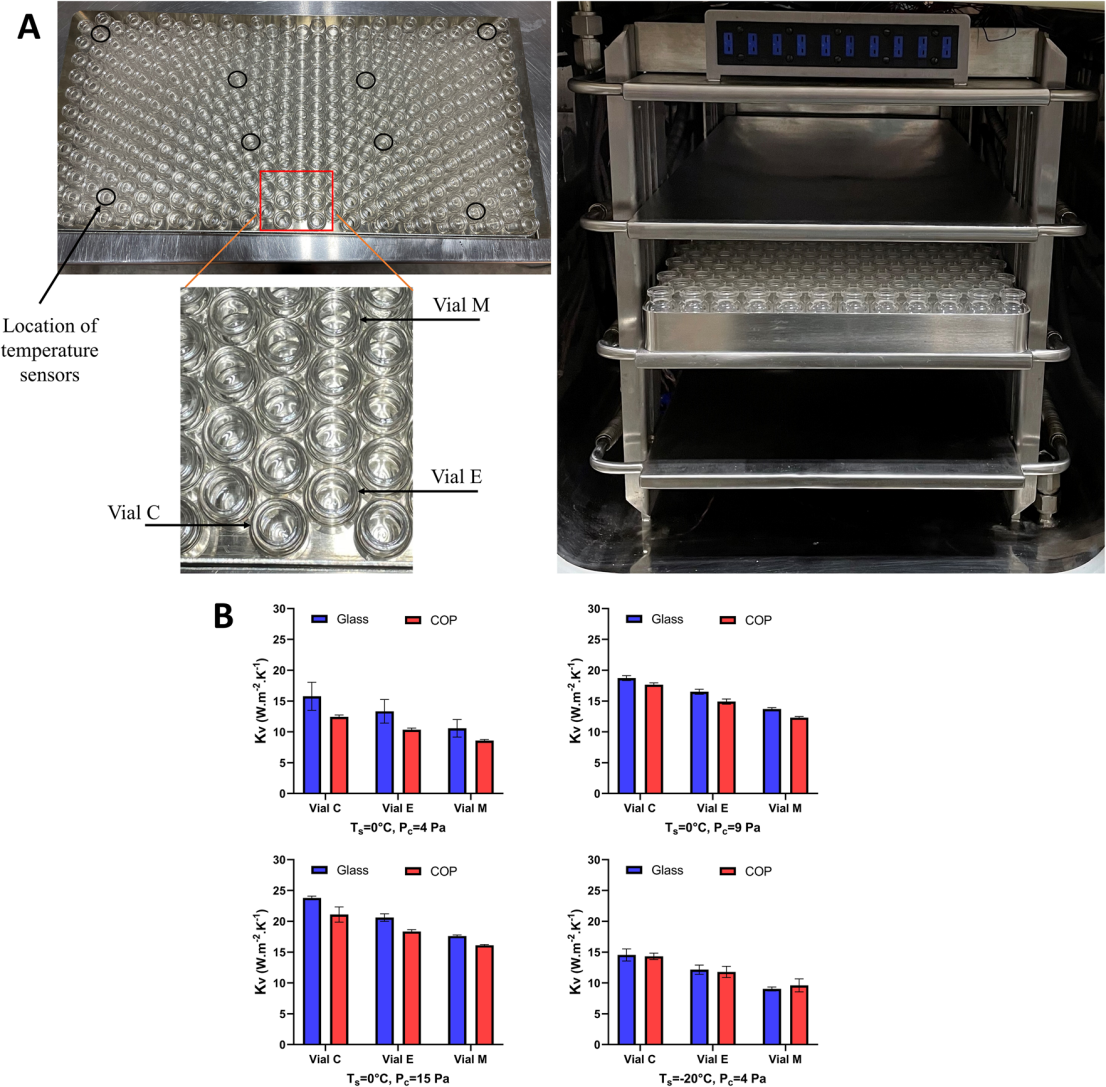
Overview of the experimental freeze-drying setup and experimental comparison of the vial total heat transfer coefficient. (**A**) Picture of 322 vials arranged on an individual freeze-drying tray and inside the shelf. (**B**) Comparison between the experimental value of heat transfer coefficient (K_v_) between hybrid COP and glass vials. Data summarizes n = 3 runs in which 322 vials were studied experimentally at various freeze-drying conditions.

The expanded model of hybrid COP or glass vials was meshed using “finer” physically optimized setting in COMSOL Multiphysics version 6.0 (Fig. 3A,B). We considered radiation, vapor conduction, and shelf conduction as three major modes of heat transfer in the model as there are reports mentioning convection can be considered negligible at low pressure related to primary drying^12,23,26^. Sources of radiation include walls, shelves, ice surface, rail, and the surface of the vials. Deionized (DI) water was considered as the model solution for freeze-drying in both simulations and experiments. A thin micron-sized layer of dense vapor termed as Knudsen layer was also considered in the model, covering all solid surfaces as reported to be essential in the literature^12,27,28^. More information regarding modeling Knudsen layer can be found in the “Materials and methods” section.

Three modes of heat flow were investigated in this study, namely, (1) contact conduction between the vials and the bottom shelf, termed as “shelf conduction”, (2) conduction between the vial and surrounding vapor, termed as “vapor conduction”, and (3) incoming radiative heat flow on the surfaces of the vials coming from the surrounding environment (e. g. surface of the wall, top-shelf, rail, and surrounding vials). The boundary conditions include (a) wall temperature imposed according to experimental measurements, (b) temperature of the top and bottom shelves imposed at 0 °C or – 20 °C, (c) chamber pressure imposed at 30, 68, or 112 mTorr depending on the simulation case, (d) temperature at the interface between the ice and vapor (T_i_). Details of boundary conditions can be found in “Materials and methods” section.

**Figure 4:**
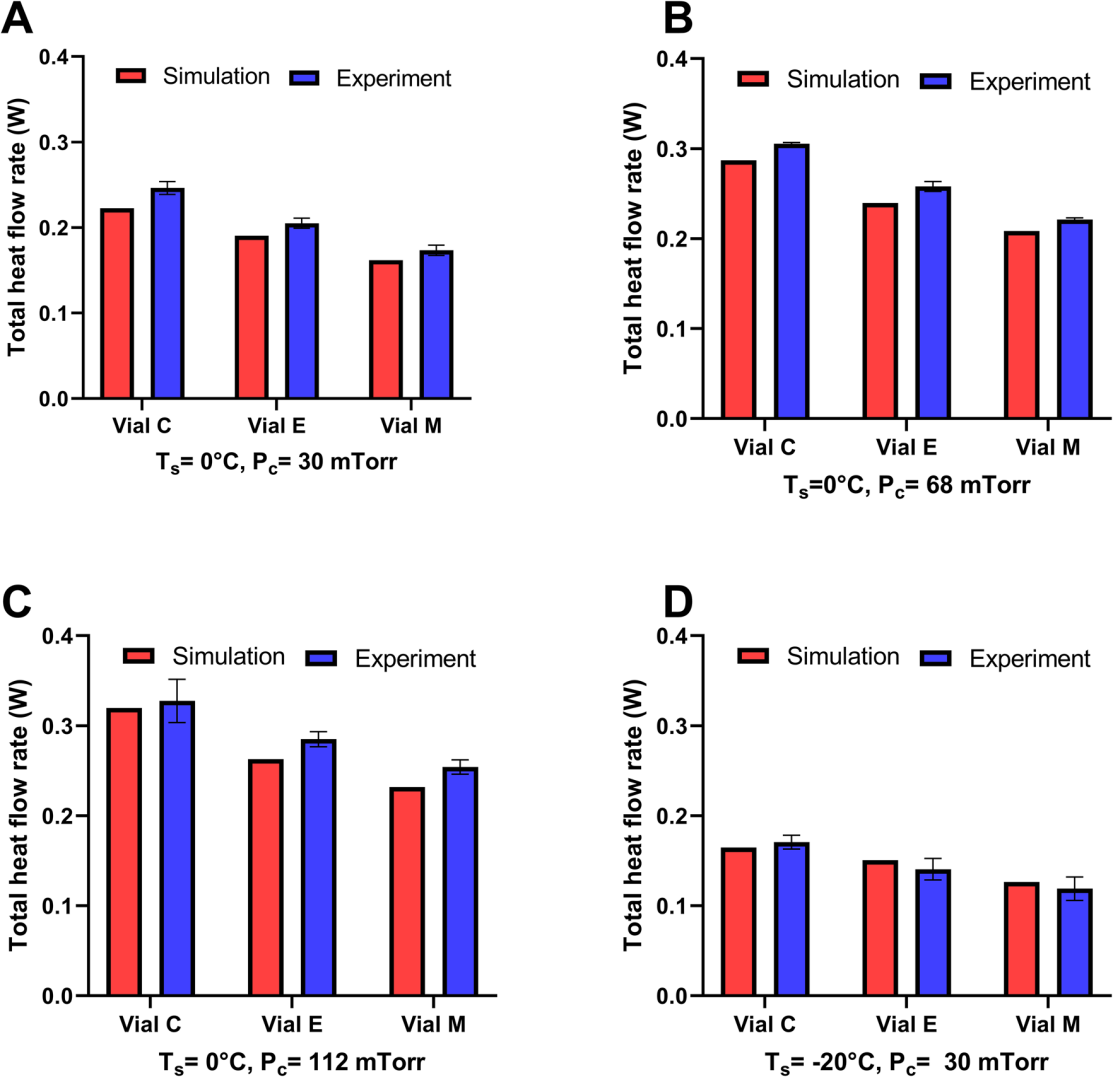
Experimental validation of the developed multiphysics model for hybrid COP vials. Comparison between the simulated and experimental values obtained for the total heat flow rate (W) regarding three types of vials studied at four different thermodynamic conditions (various T_s_ and P_c_) shown at (**A**–**D**). Error bar shows standard deviation (n = 3 runs).

**Figure 5:**
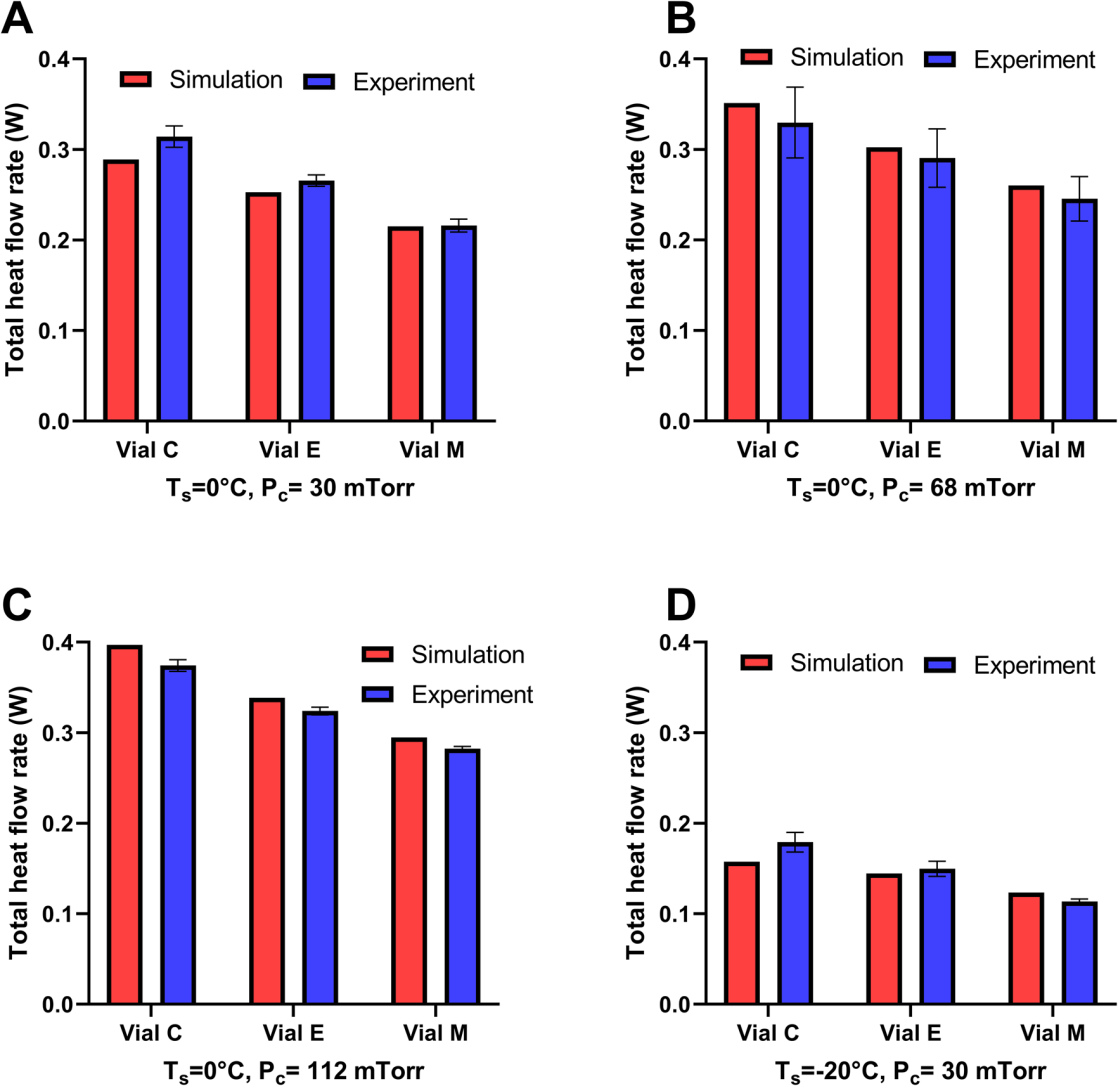
Experimental validation of the developed multiphysics model for glass vials. Comparison between the total heat flow rate obtained using simulations or experimentally related to three types of vials studied at four different thermodynamic conditions (various T_s_ and P_c_). Error bar shows standard deviation. (n = 3 runs).

As the next step, we sought to validate the numerical model by comparing the simulated and experimental heat flow rates. Total heat flow rate was considered as the summation of three modes of heat transfer considered in this study. Figures 4 and 5 compare simulated and experimental total heat flow rates at four different freeze-drying conditions regarding hybrid COP and glass vials. The percentage of difference between simulated values relative to the experimental values for hybrid COP and glass vials remained less than 10% and 8%, respectively. The average percentage of difference between simulated and experimental values for hybrid COP and glass was found to be 6.52% and 5%, respectively. These results show high capability of the numerical model in capturing the experimental conditions during steady state primary drying. At a given T_s_, the simulated heat flow rate increased by increasing chamber pressure from 30 to 112 mTorr, matching previous observations^12,28–30^. The increase was found to be up to 44% and 37% for hybrid COP and glass vials, respectively. Moreover, simulated total heat flow rate increased as the vial was located closer to the rail for both vial materials consistent with the edge vial effects reported in the literature^12,22,28^. For example, going from vial M to vial C—an increase of up to 38% and 35% in simulated heat flow rate was observed for hybrid COP and glass vials, respectively at T_s_ = 0 °C and P_c_ = 30 mTorr. This trend was relatively different in experimental flow rate where glass vial showed a slightly higher deviation as much as 3–4% going from vial M to C, compared to hybrid COP vials. Additionally, experimental heat flow rate for hybrid COP vials was found to be slightly lower than that of glass (up to 11–30% across various T_s_ and P_c_). Plotting the simulated heat flow as a function of experimental flow rates represented a strong linear correlation considering all data points for hybrid COP. We found that datapoints very well fit into a linear trend for both hybrid COP (linear regression R^2^ = 0.9821 Pearson correlation coefficient of 0.991) and glass (linear regression R^2^ = 0.9648, Pearson correlation coefficient of 0.9823) vials, validating the numerical model (Fig. S1). The Student’s t-test was also conducted between the experimental and simulated values for each vial system. The resulting p-values were found to be 0.6494 for COP and 0.9085 for glass, indicating no statistical difference between experimental and simulated values.

**Figure 6:**
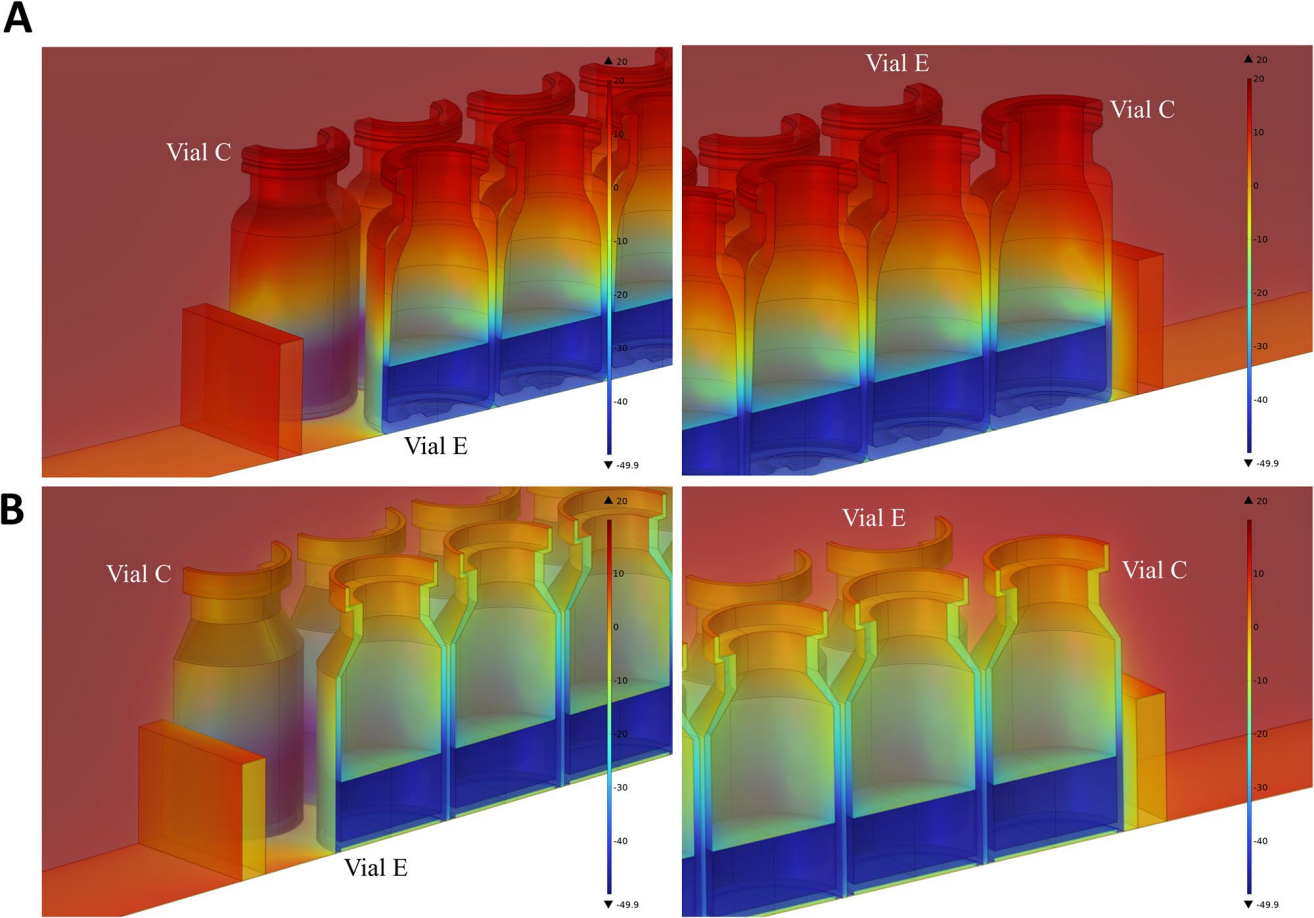
Simulated temperature contours in hybrid COP (top row) and glass vials (bottom row) during primary drying in lyophilization. Temperature contours in units of °C obtained from multi-physics simulations for (**A**) hybrid COP vials and (**B**) glass vials from different views. Shown here are the temperature contours at T_s_ = 0 °C and P_c_ = 30 mTorr.

We then investigated the temperature distribution in hybrid COP and glass vials (Fig. 6, shown for T_s_ = 0 °C and P_c_ = 30 mTorr). The ice-vapor interface had a temperature of 49.9 °C which was found to be close to a previous work reporting a temperature of 50.7 °C for borosilicate glass vials^12^. Ice temperature dropped to − 48.1 °C in glass vials and − 46.7 °C in hybrid COP vials at the interface with the bottom of the vial. Hybrid COP vials maintained a higher temperature towards top part of the vial which could be attributed to lower heat conductivity of its composition compared to glass (0.16 vs 1.1 W/m.K). This effect generated a higher temperature gradient in the body of the hybrid COP vial (around 45 °C) compared to glass vial (close to 35 °C). In glass vials, the gap between the bottom of the vial and bottom shelf was filled with vapor and Knudsen layer which generated a temperature gradient between the bottom of the vial and bottom shelf as much as 40 °C. However, in hybrid COP vials, the flat-bottom profile at the end of the vial helped decrease the temperature difference at the interface, between the vial and the bottom shelf, to half of that of glass vials, equal to approximately 20 °C. The presence of the Knudsen layer in both vial types further contributed to a larger temperature difference at the shelf-vial interface. This effect was caused due to the low thermal conductivity of the Knudsen layer compared to the vial bulk material especially at lower chamber pressure P_c_ = 30 mTorr. The temperature difference shown here for glass vials closely matched with a similar study^12^.

The heat flow arrows as a representation of conductive heat transferred from the shelf to the bottom of the vials were compared between hybrid COP and glass vials (Fig. S2). A qualitative comparison between the hybrid COP and glass vials (at identical arrow properties) represented significantly higher density of conductive heat flow arrows in the flat-bottom hybrid COP. In hybrid COP vials, the arrows were seen distributed throughout the contact interface, showing higher heat transfer through shelf conduction compared to glass vials. In glass vials, the density of heat arrows was concentrated only on the contact interface between the vial and the bottom shelf caused by presence of gap at the bottom of the vial profile (Fig. S2).

**Figure 7:**
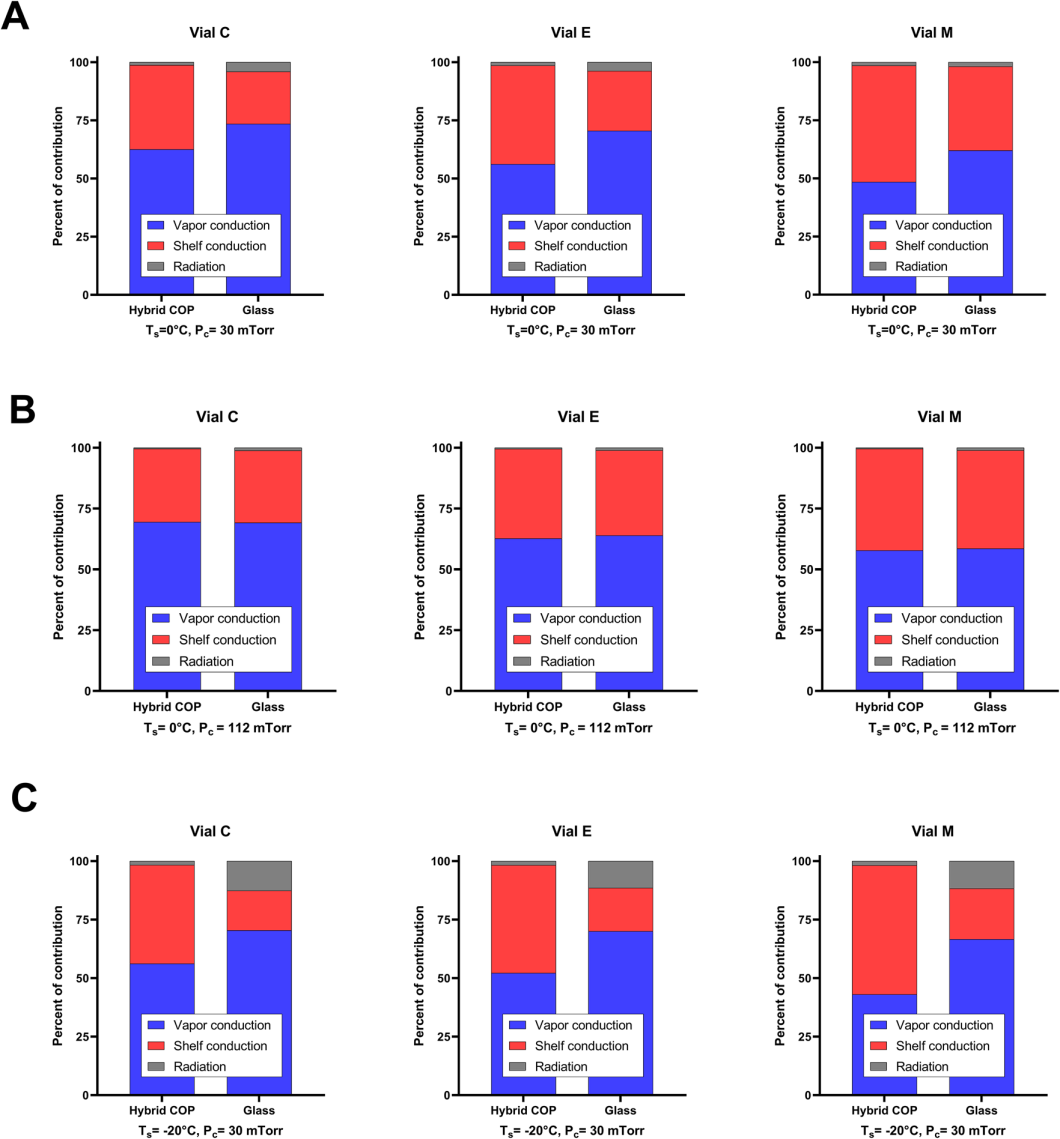
Breakdown of total heat flow rate to specific modes of heat transfer for each type of vial studied. Each graph demonstrates a comparison between hybrid COP and glass vials for vial C, vial E or vial M at different freeze-drying conditions, namely, (**A**) T_s_ = 0 °C and P_c_ = 30 mTorr, (**B**) T_s_ = 0 °C and P_c_ = 112 mTorr, and (**C**) T_s_ = − 20 °C and P_c_ = 30 mTorr.

Subsequent to study of total heat transfer, we aimed to quantify breakdown of different modes of heat transfer in each vial. We studied vapor conduction (VC), shelf conduction (SC), and radiation, as three major modes of heat transfer between the vials and the environment as shown in previous studies^12,28–30^. A breakdown of each mode of heat transfer is presented in Fig. 7, shown separately for vial C, E, and M. Results demonstrated that the contribution of each mode of heat transfer depended on P_c_, T_s_, type of the vial, and position of the vial in the tray. VC varied between 43 and 69% in hybrid COP, and within 59–73% in glass vials across various T_s_ and P_c_, making it the dominant mode of heat transfer in glass vials and most cases of hybrid COP vials.

In hybrid COP vials located as vial M, however, at lower P_c_ and T_s_ values the dominant mode transitioned towards SC, with a contribution as much as 50% and 55% for T_s_ = 0 °C and – 20 °C (P_c_ = 30 mTorr), respectively. The percentage of contribution from VC increased as vials located closer to the rail. For example, at T_s_ = 0 °C and P_c_ = 112 mTorr, VC increased by 20% and 18% in hybrid COP and glass vials, respectively, going from vial M to vial C. This could be due to higher interface with vapor in vials located as C and E compared to M which is neighbored by another 6 vials vs 3 neighboring vials for vial C, and 5 for vial E (hexagonal arrangement shown in Fig. 3). This change in the boundary conditions, experienced by edge vials, can lead to additional lateral heat flux through vapor conduction, increasing the importance of VC, as also observed in the literature^12^.

The contribution of SC was found to be higher in hybrid COP vials compared to glass vials (Fig. 7A), especially at P_c_ = 30 mTorr and T_s_ = 0 °C or T_s_ = − 20 °C. For example, at T_s_ = 0 °C, hybrid COP vials located as vials C, E, and M, had a percentage of contribution from SC which was greater than that of glass by 7%, 9%, and 10%, respectively (averaged over all chamber pressure values). Unlike VC, the percentage of contribution from SC went down as vials located closer to rail (decreased by 13% and 9% from vial C to M for hybrid COP and glass, respectively). This suggests higher significance of SC in non-edge central vial M. The absolute value of SC remained almost constant with the vial position, experiencing a change only within 0.5%, in agreement with the literature^12^. However, the contribution of SC (ratio of SC to total heat flow rate) decreased going from vial M to E and C because edge vials experienced a higher total heat flow rate caused by additional radiation and vapor conduction. The higher contribution from SC in hybrid COP vials compared to glass, especially at lower chamber pressures, matched well with the qualitative study of conductive heat flux arrows pointing out to greater shelf-mediated heat transfer in hybrid COP vials (Fig. S1).

Radiation decreased approximately by 1% and 2.8% for hybrid COP and glass vials, as a function of chamber pressure (P_c_ = 30 to 112 mTorr) matching previous reports^12,28^. Radiation was more significant in glass vials, ranging from 1.1 to 12.7% compared to 0.4 to 1.5% in hybrid COP vials across all conditions studied. As expected radiation increased as vials located closer to the rail, due to elevated radiation coming from the rail. The difference in radiation going from vial M to C in glass vials was higher compared to that of hybrid COP vials (increase up to 0.27% vs 0.94%, at T_s_ = − 20 °C), highlighting potentially higher edge effect in glass vials compared to hybrid COP vials especially at lower T_s_.

As the next step, we sought to use a parametric model of vials to study the effect of design parameters, critical during freeze-drying, on heat transfer during primary drying of hybrid COP and glass vials (Fig. S3). A parametric finite-element model was constructed with five design variables, namely, shelf-shelf distance (h_s_), vial-vial distance (d_v_), vials offset from the rail (d_c_), rail height (h_r_), and product height (h_p_). Using a design of experiment approach, we generated a table of 18 conditions with various combination of these parameters at different levels (Table S2). Each condition was then simulated separately for hybrid COP and glass vials at P_c_ of 30, 68, and 112 mTorr and at T_s_ = 0 °C.

**Figure 8:**
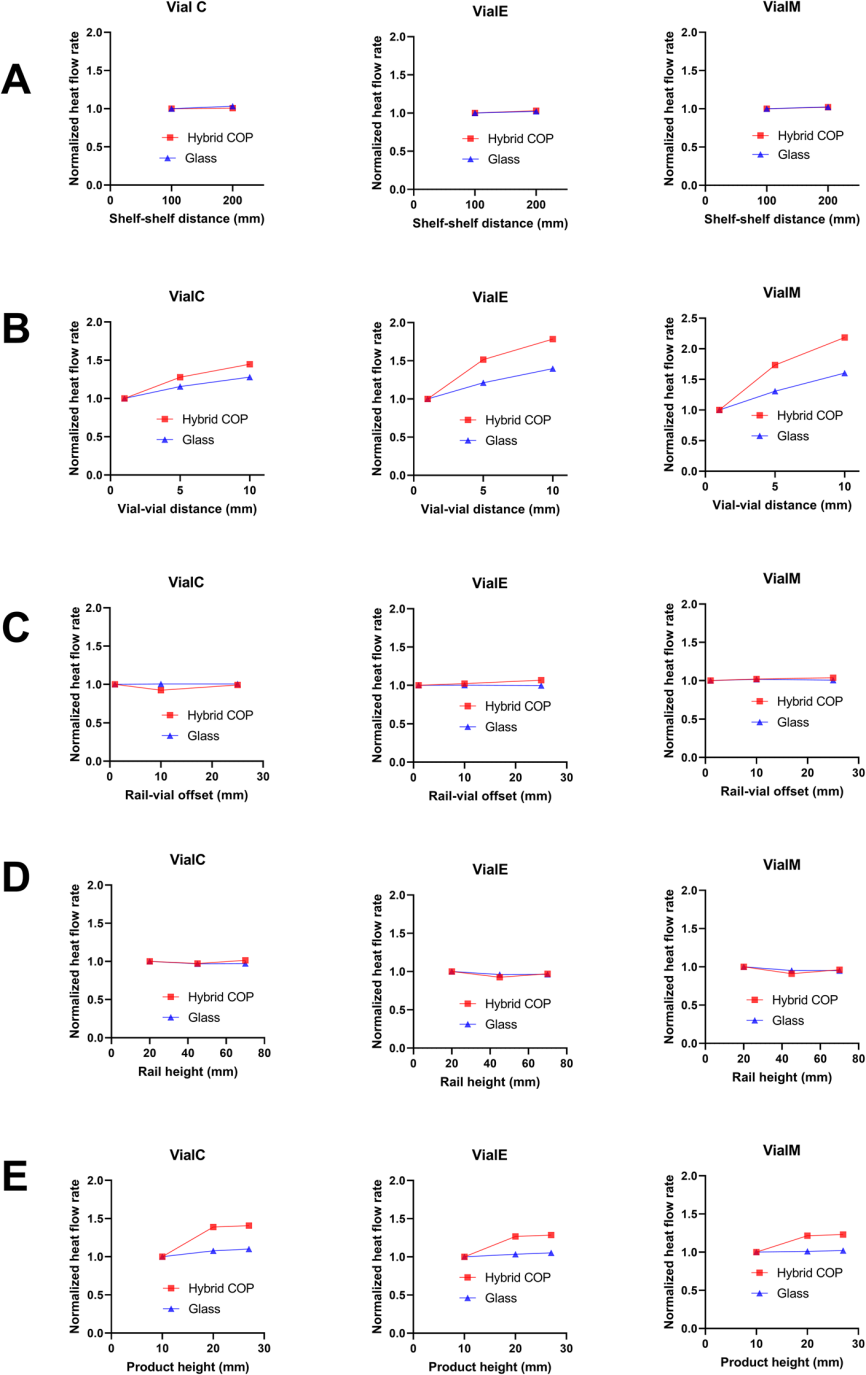
The impact of the design parameters on total heat flow rate in primary drying, comparing hybrid COP and glass vials. The y-axis in all graphs shows how the normalized heat flow rate changes as a function of freeze-drying design parameters considered in this study. Each column represents a certain type of vial, while each row corresponds to a certain design parameter. Each data point shows the averaged flow rate value for three pressures (30, 68, and 112 mTorr). Design parameters include (**A**) shelf–shelf distance, (**B**) vial–vial distance, (**C**) rail–vial offset, (**D**) height of the rail, and (**E**) height of the product during primary drying.

Figure 8 compares the normalized total heat flow rate in hybrid COP and glass vials in which each datapoint represents the averaged heat flow over the three values of P_c_. Increasing vial-vial distance (d_v_) and product height (h_p_) were found to induce the highest increase in total heat flow rate. Increasing d_v_ from 1 to 10 mm, resulted in an increase of heat flow rate up to 118% and 60% in hybrid COP and glass vials, respectively, indicating higher importance in the former. Vials located further away from the rail experienced a sharper increase in heat flow rate, representing a more pronounced effect in central vials compared to edge. Increasing d_v_ from 1 to 10 mm in hybrid COP vials increased total heat flow rate by 118%, 78%, and 44% in vial M, E, and C, respectively. The same effect was found to be less pronounced in glass vials as much as 60%, 40%, and 27%, in vials M, E, and C, respectively.

We then evaluated the impact of product height (h_p_) on heat transfer during primary drying. Increase in h_p_ led to a different effect in hybrid COP compared to glass vials. In hybrid COP vials, increasing h_p_ from 10 to 27 mm increased total heat flow rate between 23 to 41% across vial positions, while plateauing at h_p_ = 20 mm. Conversely, in glass vials, the impact of product height on heat flow rate was found rather minimal across all vial positions (maximum increase of 10% for vial C). Unlike the effect of *d*_*v*_, it was observed that the impact of h_p_ decreased as vials were located further away from the rail, consistent among both vial materials (e. g. 41% in vial C compared to 23% in vial M considering hybrid COP). The other three parameters studied resulted in a relatively less significant change in heat flow rate less than 10%, suggesting higher flexibility of these parameters from a heat transfer design perspective.

As the next step, we sought to understand the impact of the design parameters on each mode of heat transfer (Fig. S4). To this end, the heat flow rate corresponding to each mode of heat transfer was averaged over P_c_ = 30, 68, and 112 mTorr across all vial positions. In hybrid COP vials, increasing d_v_ from 1 to 10 mm increased VC and SC by 118% and 78%, respectively, while leading to a decrease in radiation as much as 1%. In glass vials, the same effect led to a less pronounced increase in VC (as much as 56%), and unlike hybrid COP vials, decreased SC and increased radiation by 4%, and 26%, respectively.

Product height (h_p_) was another parameter found to have a major effect on modes of heat transfer in both hybrid COP and glass vials. An increase in h_p_ from 10 to 27 mm increased VC up to 23% (hybrid COP) and 11% (glass). The same change increased SC in hybrid COP vials (by 28%, plateauing at h_p_ = 20 mm) but decreased it in glass vials by 18%. Radiation was found to be highly dependent on h_p_ going up by 192% and 62% in hybrid COP vials and as much as 50% in glass vials. As previous results suggested, all three modes of heat transfer remained primarily independent of shelf-shelf distance and rail-vial offset. Increasing h_r_ in hybrid COP vials decreased all three modes of heat transfer by 4–14%. In glass vials, increasing h_r_ decreased radiation by 14% (at h_r_ = 45 mm) followed by an increase up to 12% (at h_r_ = 75 mm), suggesting possibility of an optimum h_r_ in the design space.

As the last step in comparing heat transfer in hybrid COP and glass vials, we performed ANOVA and compared the percentage of contribution from each design parameter to total heat flow rate (Fig. S5A) and individual modes of heat transfer (Fig. S5B). As expected, vial-vial distance (d_v_) represented the highest percentage of contribution to the total heat flow rate. Its importance increased as the vial located further away from the rail—going from vial C, to E, and M. Product height (h_p_) was the second important parameter in both vial types, although with a more pronounced effect in hybrid COP vs glass (i.e. ranging between 9 and 48% in hybrid COP vs 0.2–14% in glass). The percentage of contribution of h_p_ increased as the vials were positioned closer to the rail as much as 39% (hybrid COP) and 14% (glass).

Subsequently, the percentage of contribution to each mode of heat transfer was studied. Vial–vial distance (d_v_) showed the highest contribution to VC in both hybrid COP and glass vials (92% and 72%, respectively). Additionally, d_v_ was found to govern SC in hybrid COP vials by having a percentage of contribution 74% greater than that of glass. Unlike hybrid COP vials, SC in glass was dominated by h_p_ with a percentage of contribution as much as 92%. Radiation was also dominated by h_p_ as evident by a percentage of contribution equal to 97% (hybrid COP) and 69% (glass) vials. In terms of VC, h_p_ was the second important parameter having a contribution percentage of 23% (hybrid COP) and 6% (glass). The other three design parameters had a contribution percentage between 0.2 and 7%.

## Discussion

In this work, we constructed a multi-physics model to compare the heat transfer during primary drying in hybrid COP vials and glass vials. We showed that multi-physics simulations can be used to successfully capture and predict the complex mechanism of heat transfer in freeze-drying not only for glass vials but also for hybrid polymeric vials. The model was used to study the total heat flow rate, temperature contour in the freeze-drying system, and the contributions coming from major modes of heat transfer during lyophilization (i.e., radiation, contact conduction, and vapor conduction). Next, the developed multi-physics model was coupled with a design of experiment approach (DoE) to systematically compare the effect of five important design parameters on the heat flow rate vials. Overall, 108 simulations were performed collectively for hybrid COP and glass vials to establish a comprehensive knowledge for this purpose.

Application of DoE in studying engineering systems has been previously shown to reduce the costs related to laborious, time-consuming and costly experiments, while providing a systematic framework for optimization^24,25,31–33^. Integration of DoE with multi-physics modeling is a relatively new approach for investigating engineering systems, and not utilized to-date for freeze-drying cycles. DOE approach can be useful for studying the effect of multiple design factors, especially when multitude of design parameters would not allow otherwise a full combinatory study. However, in the context of designing freeze-drying cycles, care must be taken toward selecting DOEs compared to an approach based on the first principles of heat and mass transfer. Accordingly, in such studies DOE can be limited to examining extremes of pressure and temperature without ever yielding a change in the product temperature.

Previous literature primarily focused on the geometry of the vials or comparing various modeling approaches to accelerate heat transfer and drying time^12,28,33,34^. These are indeed important aspects of freeze-drying, however, in large-scale freeze-drying there is typically more freedom to change the boundary conditions (e.g. vial-vial distance) than the dimensions of the vials which could require extensive manufacturing and molding re-work^14,15,35^. As a result, in this study, we focused on design parameters that are easier to modify during arrangement of vials in the freeze-drying tray, as opposed to design of the vial itself. The scope of this work was focused on analyzing the three major modes of heat transfer, namely, shelf contact conduction, vapor conduction, and radiation, during freeze-drying of vials. Future studies could incorporate other transport mechanisms (e.g. molecular level transport and heat diffusion) or a more complicated model for the sublimating solution (e.g. ice-water product with two phases).

Polymeric vials enable a more flexible design, which could be optimized for heat transfer, therefore offering new opportunities for optimization. The hybrid COP vials in this study are unique in that they benefit from a flat-bottom profile to maximize heat transfer through contact conduction with the bottom shelf. The flat-bottom led to greater shelf conduction in hybrid COP vial compared to the concave bottom profile in glass vial. In a typical glass vial, the gap between the shelf and bottom profile of the vial is filled with water vapor which can act as a resistive layer against conductive heat transfer from the bottom shelf to the bottom of the vial, a phenomenon also reported in other works^12,28^. The computational model implemented in this study demonstrated better predictability of heat flow rate in hybrid COP vials compared to glass vials as evident by a greater R^2^ for the linear regression model mapping simulated heat flow rate to experimental flow rates (R^2^_hybrid COP_ = 0.9821 vs R^2^_glass_ = 0.9648). Higher consistency of simulated heat flow rate compared to experimental flow rate can be explained from two aspects. These aspects include (a) a higher contribution of shelf conductive conduction which is more consistent across various thermodynamics conditions in freeze-dryer compared to other modes of vial heat transfer^12,28^ and (b) better dimensional tolerance in hybrid COP-fabricated vials compared to glass which is enabled by the unique manufacturing technique of hybrid COPs^1–3^.

At higher chamber pressure, the percentage of VC increased due to an increase in the conductivity of the Knudsen layer covering surfaces of the vials, which has a pressure-dependent heat conductivity. The Knudsen layer thickness is approximated to increase linearly as a function of chamber pressure (please see Eq. (3) in “Materials and methods” section). The contribution from radiation was found to be negligible compared to VC and SC in both vial compositions, decreasing at higher chamber pressure (P_c_ = 112 mTorr) similar to previous works^12,28,36^. The radiative heat flow rate was found to be higher in glass compared to hybrid COP vials, which could be related to higher total outer surface and projected surface area in glass vial compared to hybrid COP vial (approximately greater by 1 cm^2^ and 0.37 cm^2^, respectively). Radiative heat flux has been shown to lead to edge effects in vials closer to the rail (vial C and E) which receive higher radiation emitted from the wall, rail, and neighboring vials and have different boundary conditions compared to central vials^12,22,28,36,37^. The lower contribution of radiation to total heat flow in hybrid COP vial compared to glass can further explain higher consistency of heat transfer coefficient (i.e., lower STD) in hybrid COP vials measured experimentally (Fig. 3) as also reported previously^3^.

Two design parameters, namely, vial-vial distance and drug product height were found to play a major role in the heat flow rate consistency among hybrid COP and glass vials. The heat flow rate was found to be mostly independent of other parameters studied (i.e. shelf-shelf distance, rail-vial offset, and the rail height). This observation can provide some design flexibility for arrangement of vials within industrial scale freeze-drying trays. For example, freeze-drying trays can be placed reasonably closer to each other (vertically) in the chamber without concerns related to changing the freeze-dried product quality or drying time. Packing more trays within the limited chamber space can increase the throughput and reduce operational costs. Moreover, the rail height was found to have no significant effect on heat transfer, giving more design customizability for the rail especially in view of automating handling of vials. Independence of heat flow rate from vial–rail distance indicates potentially more vials can be packed inside the freeze-drying tray given a certain space.

An effective strategy for increasing heat flow rate in vials was to increase the vial–vial distance and product height. Increased vial–vial distance was found to mostly contribute to higher conduction mediated by the vapor surrounding the vials (VC). Higher vial–vial distance can potentially allow easier access of the surrounding vapor to the outer surface of the vials to accelerate heat transfer across both hybrid COP and glass vials. The effect is more pronounced in central vials which have a more limited space around to interact with vapor, due to the presence of other neighboring vials.

The effect of vial–vial distance on the total heat flow rate can also be explained in view of the endothermic nature of the sublimation process. Each sublimating vial acts as a heat sink for the surrounding vials, therefore reducing the total heat influx towards the vials in vicinity. Increasing the vial–vial distance places the sublimating vials further away from each other and therefore collectively reduces the cooling effect of surrounding vials impacting a given vial. This effect can increase the total heat influx as a function of vial-vial distance as also shown in Fig. 8B and Fig. S4B. A similar rationale can be used to explain higher total heat flow rates in edge vials compared to central vials. Edge vials are surrounded by a fewer number of vials compared to central vials. As a result, the cooling effect of surrounding vials on edge vials during primary drying is going to be less than on central vials. This leads to a higher heat influx by edge vials compared to central vials, commonly known as the “edge vial effect” demonstrated in this work and others^12,22,28^.

The exact effect of product height (h_p_) and vial-vial distance (d_v_) on heat flow rate in vials during freeze-drying is rarely understood. We hypothesize that increasing the product height (h_p_) will increase the surface area of the cold sublimating ice with the vial containing it. As a result, the average temperature will drop in the vial. For the surrounding vapor at a given temperature, decreased temperature of the vial would lead to a higher temperature gradient between the vial and the surrounding vapor. Since vapor conduction is dependent on the temperature gradient between the vial and the surrounding vapor, vapor conduction between the vial and the vapor (VC) will ultimately increase.

The same phenomenon is applicable to describe changes in SC, such that decrease in the vial temperature, as a result of higher ice height (h_p_), would further decrease the temperature at the vial base contacting the bottom shelf. For a given T_S_, this would lead to a higher temperature gradient between the shelf and the vial. This higher temperature gradient between the bottom shelf and the vial base would lead to a higher shelf conduction (SC). In comparing glass and COP, it should be noted that due to lower thermal conductivity of COP, a higher temperature gradient can be generated throughout the bulk of the polymeric vial. This effect in addition to flat-bottom profile at the base of COP vials would lead to a more pronounced increase in both VC and SC in COP compared to glass. In glass vials, increased h_p_ had a relatively minor impact on VC and SC which can be attributed to (1) lower temperature gradient throughout the bulk of the glass vials and (2) lower surface area between the vial bottom and the bottom shelf. In glass vials, the contribution of VC slightly decreased showing that change in the temperature gradient was not a major factor. This was because the high thermal conductivity in glass vials reduced the impact of the increased temperature gradient (therefore not much change in VC).

Regarding the dependence of SC on d_v_, increasing d_v_ will position vials further away from the freeze-drying wall which has the highest temperature in simulations. This condition will surround the vials with colder vapor which ultimately decreases the average vial temperature. Decreased temperature in vials will lead to a greater temperature gradient for a given T_S_. This is further supported by results from Fig. 8B, showing that vial M has a greater increase (almost twofold) in the total heat transfer while the vials positioned closer to the freeze-drying wall (vials E and C) experienced a less pronounced increase in their energy.

Since shelf conduction is directly proportional to this temperature gradient, SC between the vial and bottom shelf is expected to increase. This is more pronounced in COP vials because of its lower thermal conductivity compared to glass, yielding a greater temperature gradient in COP vials. Moreover, the effect of SC in COP vials is elevated due to having a greater interfacial surface with the bottom shelf. On the other hand, the impact of d_v_ on SC in glass vials is rather minor due to its higher thermal conductivity compared to COP. Similar to the effect of h_p_, this feature along with small interface between the glass vials and the bottom shelf reduces the importance of SC in glass vials.

Increasing drug product fill volume or height was another strategy found effective to increase heat flow rate, especially in hybrid COP vials. Elevated drug product height more notably increased radiation in hybrid COP and both radiation and vapor conduction in glass vials. This is especially a critical consideration in choosing vials as hybrid COP vials can handle a higher volume fill thanks to resistance to breakage during freezing, compared to glass vials^3,17–20^. Simulations herein demonstrate freeze-dried solutions with higher fill-volume can benefit from a higher heat flow with hybrid COP vials.

## Conclusions

To the best of our knowledge, this is the first comparative study of the heat transfer behavior between hybrid COP vials and glass vials for freeze-drying applications. Multi-physics finite-element modeling was successfully used to simulate heat transfer behavior during freeze-drying of hybrid COP and glass vials. Simulated heat flow rate was found in excellent agreement (less than 10% of error) with experimental values. Hybrid COP vials represented a higher predictability with the computational model which can be attributed to better dimensional consistency in hybrid COP vials as well as higher contribution of shelf heat conduction in hybrid COP vials. This suggests higher product and process consistency in hybrid COP vials compared to glass especially at lower chamber pressures (~ 30 mTorr). By integrating a DoE approach with simulations, we were able to construct a parametric model of the vials arranged in a freeze-drying tray. Results demonstrated that simple strategies such as increasing vial–vial distance in hybrid COP and glass vials and increasing drug product height in hybrid COP vials can increase the heat flow rate, thus decreasing the primary drying time. Increasing drug product height is especially preferable in hybrid COP vials due to their resistance against breakage at high fill-volume compared to glass vials, reducing the necessary vial size as a result. We further demonstrated that the dominant mode of heat transfer in flat-bottom hybrid COP vials depended on the freeze-drying conditions. More specifically, heat transfer tended to transition from vapor conduction to shelf conduction at lower chamber pressure (i.e. 30 mTorr) and shelf temperature (i.e. – 20 °C). Heat transfer in glass vials was predominantly governed by vapor conduction. The flat-bottom of the hybrid COP vials enhanced heat conduction with the bottom shelf and led to a higher density of conductive heat flux. Further, hybrid COP vials exhibited more consistent heat flow rate and total heat transfer coefficient across a group of vials in the freeze-drying tray. Results of this study can contribute to design optimization of vials regarding both material properties and geometry, as well as arrangement of vials in freeze-drying tray for improved drying rate and increased product consistency. Future work can be related to modeling novel techniques to improve heat transfer in vials such as simulation of forced convection in the freeze-drying chamber or various temperature distribution along the bottom shelf and its impact on heat transfer in vials.

## Materials and methods

### Multi-physics simulations

Multiphysics simulations were conducted in COMSOL Multiphysics V 6.0 using Heat Transfer in Solids and Fluids module. Simulations were run on a PC (Windows 10 Pro) equipped with an Intel^®^ Xeon^®^—W-1290 at 3.70 GHz with a 16-core processor and 32 GB of RAM. Each simulation lasted for approximately 30 min. A “Finer” mesh setting was used in all simulations. Simulations were conducted at steady state using PARDISO solver (https://pardiso-project.org/) at a relative tolerance of 10^–4^. The number of mesh elements generated varied between models but was approximately between 1.1 to 1.7 million elements composed of Tetrahedra elements.

The temperature of the upper and bottom shelves was imposed as boundary conditions (T_s_). Temperature of the wall was measured experimentally, while temperature at the vapor-ice interface (${T}_{i}$) was calculated using the Clausius-Clapeyron Relationship described as follows^35^:

1 $${T}_{i}=\frac{6139.6}{28.8912-\mathrm{ln}({P}_{i})}.$$

In which, *P*_*i*_ was assumed to be same as the chamber pressure *P*_*c*_ and *T*_*i*_ is the vapor-ice interface temperature. It was assumed that there is no mass transfer between the vapor-ice interface and the chamber vapor, resulting in *P*_*i*_ ~ *P*_*c*_.

### Heat transfer by conduction

Conductive heat flux within a given object was calculated using the first Fourier law described below^39,40^:

2 $$\overrightarrow{{q}_{c}}=-\lambda \nabla T,$$

where ${q}_{c}$ is the conductive heat flux, $\lambda$ is the thermal conductivity of the material (Table S1) and $\nabla T$ is the temperature gradient within an object.

In this study, due to low pressure levels simulated (30–112 mTorr), it was assumed that the dominant mode is free-molecular patch or Knudsen regimen^12,26,27^. The heat conductivity of Knudsen layer covering all solid surfaces simulated was calculated from the following formula driven from elegant work of Scutella et al.^12^:

3 $${\lambda }_{kn}=2\alpha {\wedge }_{0}{P}_{c}{l}_{kn}.$$

In which $\alpha$ is an empirical constant representing the quality of energy exchange at solid–gas interface which is calculated from the K_v_–P_c_ relationship obtained from sublimation experiments (see the section “Experimental”). Parameter ${\wedge }_{0}$ represents the heat transfer coefficient for the free molecular flow which is equal to 1.99 $\frac{W}{{K Pa m}^{2}}$. Parameter *P*_*c*_ is the chamber pressure and ${l}_{kn}$ is the thickness of the Knudsen layer (${l}_{kn}$) which was chosen as 150 µm and 90 µm for hybrid COP and glass vials, respectively.

Heat flux through contact conduction between two objects in contact is defined as^26^:

4 $${q}_{cc}={K}_{cc }\left({T}_{1}-{T}_{2}\right).$$

In which ${q}_{cc}$ is the conductive flux from object 1 to object 2, *K*_*cc*_ is the contact conduction heat transfer coefficient between objects 1 and 2, which depends on the quality of the contact between the objects. Additionally, *T*_1_ and *T*_2_ are the absolute temperatures at the contact surface of object 1 and object 2, respectively. In this study, two types of contact were considered, namely, contact between vial and bottom shelf, and contact between rail and bottom shelf. The heat transfer coefficient for contact conduction between rail and bottom-shelf (*K*_*cc,r*_) was selected from the literature. The heat transfer coefficient for contact conduction between vials and bottom shelf (*K*_*cc,v*_) was calculated from experimental data as explained below:

First, sublimation experiments were conducted at various chamber pressures to obtain the total heat transfer coefficient of the vial ${K}_{v}$ described as^12,26–28,41^:

5 $${K}_{v}=\frac{\Delta H\dot{m}}{{A}_{b}({T}_{s}-{T}_{b})}.$$

In which ${T}_{b}$ represents the absolute temperature at the bottom end of the product inside the vial, *A*_*b*_ is the outer surface area of the vial bottom end, *T*_*s*_ is the absolute shelf temperature, $\Delta H$ is the latent heat of ice sublimation ($\Delta H=2.8\times {10}^{6} \mathrm{J}/\mathrm{kg}$), and $\dot{m}$ represents sublimation rate. Calculation of ${K}_{v}$ and $\dot{m}$ can be done experimentally (see “Experimental” section below).

The total heat transfer coefficient for vials can be written as^26–28,37,41^:

6 $${{K}_{v}={K}_{c}+K}_{r}+{K}_{g}.$$

In the equation above, ${K}_{c}$, ${K}_{r}$, and ${K}_{g}$ represent heat transfer coefficient by contact conduction with the bottom shelf, radiation, and vapor conduction, respectively. Of these, only ${K}_{g}$ depends on the chamber pressure as follows^26–29,41^.

7 $${K}_{g}=\frac{{C}_{2}{P}_{c}}{1+\frac{l}{{\lambda }_{amb}}{C}_{2}{P}_{c}}.$$

In which P_c_ is the chamber pressure, ${\lambda }_{amb}$ is the molecular conductivity of water vapor at ambient pressure equal to 0.025 $\frac{W}{m K}$. Parameter *l* represents effective distance characterizing the gap between the bottom shelf and bottom of the vial. Parameter *C*_*2*_ can be calculated as^22,28,41^:

8 $${C}_{2}={\wedge }_{0}\frac{{\alpha }_{c}}{2-{\alpha }_{c}}{(\frac{273.15}{{T}_{gas}})}^{0.5}.$$

In which, ${\alpha }_{c}$ is accommodation coefficient (equal to 0.48 for water vapor^28^), and *T*_*gas*_ is the average temperature between the product at the sublimation interface (*T*_*i*_) and the shelf temperature (*T*_*s*_). Accordingly, the summation of ${K}_{c}+{K}_{r}$ can be found by curve-fitting the relevant mathematical function to the graph of ${K}_{v}$ as a function of ${P}_{c}$, obtained experimentally at *P*_*c*_ = 30, 68, and 112 mTorr. After obtaining *C*_2_, the coefficient of *α*, used to estimate thermal conductivity of Knudsen layer, can be calculated as^12,28^:

9 $$\alpha =\frac{{C}_{2}}{{\wedge }_{0}}.$$

Parameter $\alpha$ for hybrid COP and glass vials was found to be 0.34 and 0.33 (dimensionless). Additionally, it can be shown that ${K}_{r}$ can be independently approximated as follows^26,28,41^.

10 $${K}_{r}=\mathcal{F}\sigma \left({T}_{S}+{T}_{b}\right)\left({{T}_{S}}^{2}+{{T}_{b}}^{2}\right).$$

In which $\sigma$ is Stefan–Boltzmann constant and $\mathcal{F}$ is visualization factor which can be calculated as^26–28^:

11 $$\mathcal{F}={\mathcal{F}}_{top}+{\mathcal{F}}_{bottom}.$$

In which ${\mathcal{F}}_{top}$ is visualization factor between top of the vial and the top shelf (related to radiation between vial and top shelf) and ${\mathcal{F}}_{bottom}$ is visualization factor between the bottom of the vial and the bottom shelf (related to radiation influx from the bottom shelf to vial). These visualization factors can be approximated as follows^26–28^:

12 $${\mathcal{F}}_{b}=\frac{1}{1+\left(\frac{1}{{e}_{v}}-1\right)+\left(\frac{1}{{e}_{s}}-1\right)},$$

13 $${\mathcal{F}}_{top}={e}_{v}.$$

In which ${e}_{v}$ is emissivity of vial and ${e}_{s}$ is emissivity of the shelf (see Table S1). By calculating ${K}_{r}$ independently from Eq. (10) and calculating ${K}_{c}+{K}_{r}$ from curve-fitting, ${K}_{c}$ can be obtained. There are only a few studies available related to what specifically determines *K*_*c*_^9,28,42,43^. As reported in the literature, ${K}_{c}$ is only dependent on the type of contact between vial and the bottom shelf such that *K*_*c*_ is linearly dependent on the contact surface area as^9,28,42,43^:

14 $${K}_{c}={C}_{1}{A}_{c}.$$

In which C_1_ is an empirical coefficient and A_c_ is the area of the contact surface between the vial and bottom shelf. The *K*_*c*_ obtained above corresponds to the entire vial surface area. Since in the model only a portion of the vial bottom surface is in contact with the bottom shelf, the contact conduction coefficient was calculated as follows:

15 $${K{\prime}}_{cc,v}={K}_{cc,v}\frac{{S}_{f}}{{S}_{c}}.$$

In which ${K{\prime}}_{cc,v}$ is the effective contact conduction heat transfer coefficient used for vials in the simulations (Table S1). S_f_ corresponds to the surface area of the entire vial bottom and *S*_*m*_ represents the surface area in contact with the bottom shelf in the simulations. The ratio of $\frac{{S}_{f}}{{S}_{m}}$ for hybrid COP and glass vials were calculated as 2.18 and 6.28, respectively. To calculate the K_CC_ coefficients, the following procedure was followed as fully described in Ref.^26^:

1. Calculation of total heat transfer coefficient for vial (K_v_) at various chamber pressures.
2. Calculation of the intercept of K_v_–P_c_ plot at P_c_ = 0, by curve-fitting. This will yield K_r_ + K_c_.
3. Direct calculation of K_r_ based on mathematical formulas in the literature.
4. Calculation of K_c_ after obtaining K_c_ + K_r_ from step 2 and K_r_ from step 3.
5. Calculation of the effective coefficient of contact conduction by multiplying the resulting K_c_ from step 4 by the ratio of (S_f_/S_m_). S_f_ corresponds to the surface area of the entire vial bottom and S_m_ represents the surface area in contact with the bottom shelf in the simulations.

Resulting values are reported in Table S1.

### Simulation of radiation physics

Radiation heat transfer was simulated using Surface-to-Surface Radiation module based on Hemicube method at a surface-to-surface radiation resolution of 256. Radiation module and Heat Transfer in Solids and Fluids were coupled using Heat Transfer with Surface-to-Surface Radiation. Radiative heat flux was modeled using Stefan–Boltzmann equation^24,25,37,38^, described below:

16 $${q}_{r}={F}_{1\to 2}\sigma \left({T}_{1}^{4}-{T}_{2}^{4}\right).$$

In which ${q}_{r}$ is the radiative heat flux from surface 1 to surface 2, ${F}_{1\to 2}$ is the visualization factor between surface 1 and surface 2, sigma is the Stefan-Boltzmann constant. T_1_ and T_2_ are the absolute temperatures of surface 1 and surface 2, respectively. Surface-to-surface radiation model in COMSOL V 6.0 is a powerful technique which incorporates shadowing effect and automatically calculates the shape factor between each pair of surfaces. In this model, following assumptions were made based on previous studies^26,27,40,41^:

1. Radiation in the low-pressure (Knudsen) regimen in the freeze-drying chamber is non-negligible.
2. Solid surfaces were considered opaque.
3. Radiation and absorption occur in the same spectral range.
4. Radiation coming from low-pressure vapor inside the freeze-drying chamber is negligible.

Equations (10–13) were used to calculate view factor for vial M, therefore calculating K_r_ and ultimately K_CC_ from experiments to be used for simulations. The calculation of view factors in simulations was carried out using COMSOL Multiphysics built-in Hemicube method which takes into account any changes in the geometry. This method automatically updates changes in the geometry arrangements within the model and updates the view factors accordingly using the updated mesh. The method Hemicube has been previously used and compared with experimental data in the literature, showing very good agreement and prediction power^44,45^. This method analyzes the view factor of the face of each element in the mesh by rendering digital images of the geometry in five directions^46^. A full description of the method used to calculate view factors using Hemicube method can be found in Refs.^46,47^. Emissivity was estimated based on available data in the literature, namely^48^ for COP and^12^ for glass.

### Design of experiment analysis

Design of experiment creation and ANOVA were performed in Minitab as shown in Table S2. All DoE simulations were repeated for three chamber pressure values (30, 68, and 112 mTorr). DoE results of heat flow rate were accordingly averaged over these pressure values. The Student’s t-test was performed in GraphPad Prism.

### Experimental freeze-drying

Freeze-drying experiments were performed on a pilot freeze-dryer (Millrock Technology REVO RV85) with 4 shelves. Vials were positioned on a tray placed on the second shelf from bottom (Fig. 3A). The distance between the shelf and the wall was 79 mm and the distance between two adjacent shelves was 89 mm. Each tray (bottomless) had a dimension of 610 mm by 305 mm. Vials seating on the tray were surrounded by a stainless-steel rail (rail model here) and directly in contact with the bottom shelf. In freeze-drying runs, 8 temperature sensors (Type T, OMEGA Engineering) were placed on specific vials (shown in Fig. 3A) to monitor the temperature of the vials during freeze-drying cycles. All temperature values used for analysis were averaged within 30 min of steady state conditions, taking place about 125 min to 155 min after the start of the primary drying. During this period, the actual shelf temperature and actual chamber pressure reach the set shelf temperature and chamber pressure, respectively. Additionally, in this period, changes in the shelf temperature and chamber pressure remain negligible (less than 3%) and can be considered almost constant. All comparisons considered in this study refer to this period. This approach based on local steady state conditions has also been used successfully in the literature showing good agreement with modeling practices^12^. The transient phase in which the temperature ramped up to the set shelf temperature was excluded from analysis. The primary drying step lasted for approximately 6–7 h.

The experimental sublimation heat flow was determined gravimetrically. The initial and final mass of each vial was weighted using a balance (Scout Pro SP202, OHAUS Corporation). Subsequently, the mass flow rate ($\dot{J}$) after sublimation cycles for each vial was calculated as^12,23,24,38,41^:

17 $$\dot{J}=\frac{{m}_{i}-{m}_{f}}{\Delta t}.$$

In which ${m}_{i}$ and ${m}_{f}$ correspond to masses of the vial, at the start and end of the sublimation. Furthermore, $\Delta t$ is the duration of time for steady state sublimation measured after reaching steady state up to the end of the primary drying. Experimental heat flow rate was then measured as follows:

18 $$\dot{Q}=\Delta H\dot{J}.$$

In which $\Delta H$ is the latent heat of ice sublimation equal to 2.8 × 10^6^ J/kg. The relationship described above is valid under the assumption that sublimation is in a pseudo-stationary state as reported in the literature^12,26,27,38,41^. Both experiments and simulations used the same boundary conditions and dimensions of the shelf relative to wall and shelf-to-shelf vertical distance. Glass vials (Schott, 10 mL-nominal volume) were filled with 5.0 mL of deionized water reaching a height of 11 mm in the vial. Hybrid COP vials (SiO_2_ Medical Product, 10 mL nominal volume) with similar dimension of outer diameter and vial height were used for comparisons. The vials were modeled with minor simplifications to reduce mesh complexities. Dimensions of the vials were obtained from technical drawings of the vials.

Chamber pressure during freeze-drying experiments were measured and controlled (± 2 mTorr) using a capacitance manometer (622C, MKS Instruments) and the temperature was measured using temperature sensors positioned at the bottom center of the selected vials. Four different freeze-drying experiments (n = 3 per experiment) were performed to validate the multi-physics models developed for hybrid COP or glass vials. In three experiments, shelf temperature was set at 0 °C while the chamber pressure varied at 4, 9, and 15 Pa. In one experiment, the shelf temperature was set at – 20 °C and the chamber pressure was set at 4 Pa. In each experiment, a number of 322 Vials (either hybrid COP or glass made) were positioned in the middle shelf of the freeze-drying equipment. The vials were arranged in a hexagonal arrangement following the method described in Refs.^12,16^. During each freeze-drying run, vials were pre-cooled at 1 °C/min and underwent a freezing step of at least four hours, the chamber pressure decreased, and the shelf temperature increased at a ballistic rate, as quickly as achievable by the lyophilizer, to the designated shelf temperature (T_s_). Freeze-drying cycles continued until approximately 30% of the water initial mass was sublimated. There are various reports as how many rows of vials away from the rail are affected by the “edge effect” caused by presence of the rail but generally the effect has been shown to be dominant in the 2nd and 3rd row vials^12,28,41,49^. Therefore, in this study we considered vials located at the 8th row as the central vials to be well further away from the edge vials.

### Supplementary Information

Supplementary Information.

## Data Availability

Data necessary to replicate this study is mostly presented throughout the manuscript. Raw data can be provided by contacting MS upon reasonable request.
