# Supplementary material for: A comparative study of freeze-drying heat transfer in polymeric vials and glass vials
Source: Sci Rep. 2023 Oct 23;13:18092. doi: 10.1038/s41598-023-40777-3 (PMC10593743; doi:10.1038/s41598-023-40777-3)
Supplement: Supplementary file 1 — Supplementary Information. [file 41598_2023_40777_MOESM1_ESM.docx]

A Comparative Study of Freeze-Drying Heat Transfer in Polymeric Vials and Glass Vials

Morteza Sarmadi^1^ , Spencer Holmes^2^, Royal Agha^2^, Brandon Davenport^2^, Christopher Weikart^1^, T.N. Thompson^2^

^1^ SiO_2_ Medical Products, Auburn, AL, USA, 36830

^2^ Millrock Technology, Kingston, NY, USA, 12401

**Supplemental files**

**Tables**

**Table S1.** Thermal properties used in the model.

| **Parameter Description** | **Value** | **Unit** |
| --- | --- | --- |
| Thermal conductivity of glass vial | 1.1 | W/m·K |
| Thermal conductivity of hybrid COP vial | 0.16 | W/m·K |
| Thermal conductivity of the rail | 16.5 | W/m·K |
| Thermal conductivity of ice | 2.2 | W/m·K |
| Thermal conductivity of the vapor at atmospheric pressure | 2.5×10^-2^ | W/m·K |
| Effective heat transfer coefficient of contact conduction between rail and bottom shelf | 10 | W/m^2^·K |
| Effective heat transfer coefficient of contact conduction between glass vial and bottom shelf | 28.95 | W/m^2^·K |
| Effective heat transfer coefficient of contact conduction between hybrid COP vial and bottom shelf | 3.82 | W/m^2^·K |
| Emissivity of the ice | 0.98 | Unitless |
| Emissivity of rail (stainless steel) | 0.14 | Unitless |
| Emissivity of shelf (stainless steel) | 0.18 | Unitless |
| Emissivity of glass vial | 0.78 | Unitless |
| Emissivity of hybrid COP vial | 0.95 | Unitless |
| Emissivity of the wall | 0.13 | Unitless |

**Table S2.** Table of design of experiment used in this study generated based on an L_18_ orthogonal array.

| Simulation No. | h_s_ (mm) | d_v_ (mm) | d_c_ (mm) | h_r_ (mm) | h_p_ (mm) |
| --- | --- | --- | --- | --- | --- |
| 1 | 100 | 1 | 1 | 20 | 10 |
| 2 | 100 | 1 | 10 | 45 | 20 |
| 3 | 100 | 1 | 25 | 70 | 27 |
| 4 | 100 | 5 | 1 | 20 | 20 |
| 5 | 100 | 5 | 10 | 45 | 27 |
| 6 | 100 | 5 | 25 | 70 | 10 |
| 7 | 100 | 10 | 1 | 45 | 10 |
| 8 | 100 | 10 | 10 | 70 | 20 |
| 9 | 100 | 10 | 25 | 20 | 27 |
| 10 | 200 | 1 | 1 | 70 | 27 |
| 11 | 200 | 1 | 10 | 20 | 10 |
| 11 | 200 | 1 | 10 | 20 | 10 |
| 13 | 200 | 5 | 1 | 45 | 27 |
| 14 | 200 | 5 | 10 | 70 | 10 |
| 15 | 200 | 5 | 25 | 20 | 20 |
| 16 | 200 | 10 | 1 | 70 | 20 |
| 17 | 200 | 10 | 10 | 20 | 27 |
| 18 | 200 | 10 | 25 | 45 | 10 |

**Supplemental Figures**


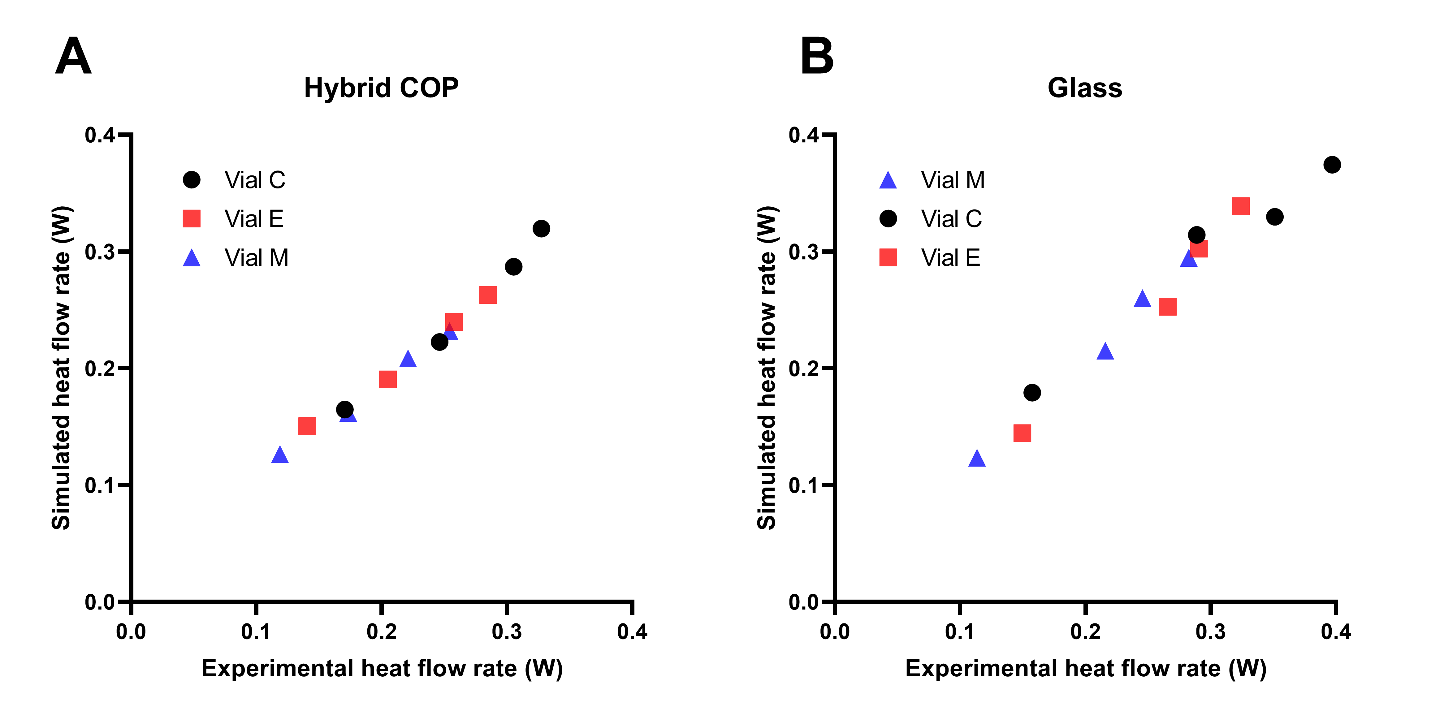


**Fig. S1 Scatter plot of simulated vs experimental heat flow rates.**Data shown for A) hybrid COP and B) glass vials plotted for various vial types/freeze-drying conditions. The R^2^ of fitted line, mapping simulated values to corresponding experimental values, for hybrid COP and glass is equal to R^2^_COP_= 0.9821 and R^2^_glass_= 0.9648, respectively.


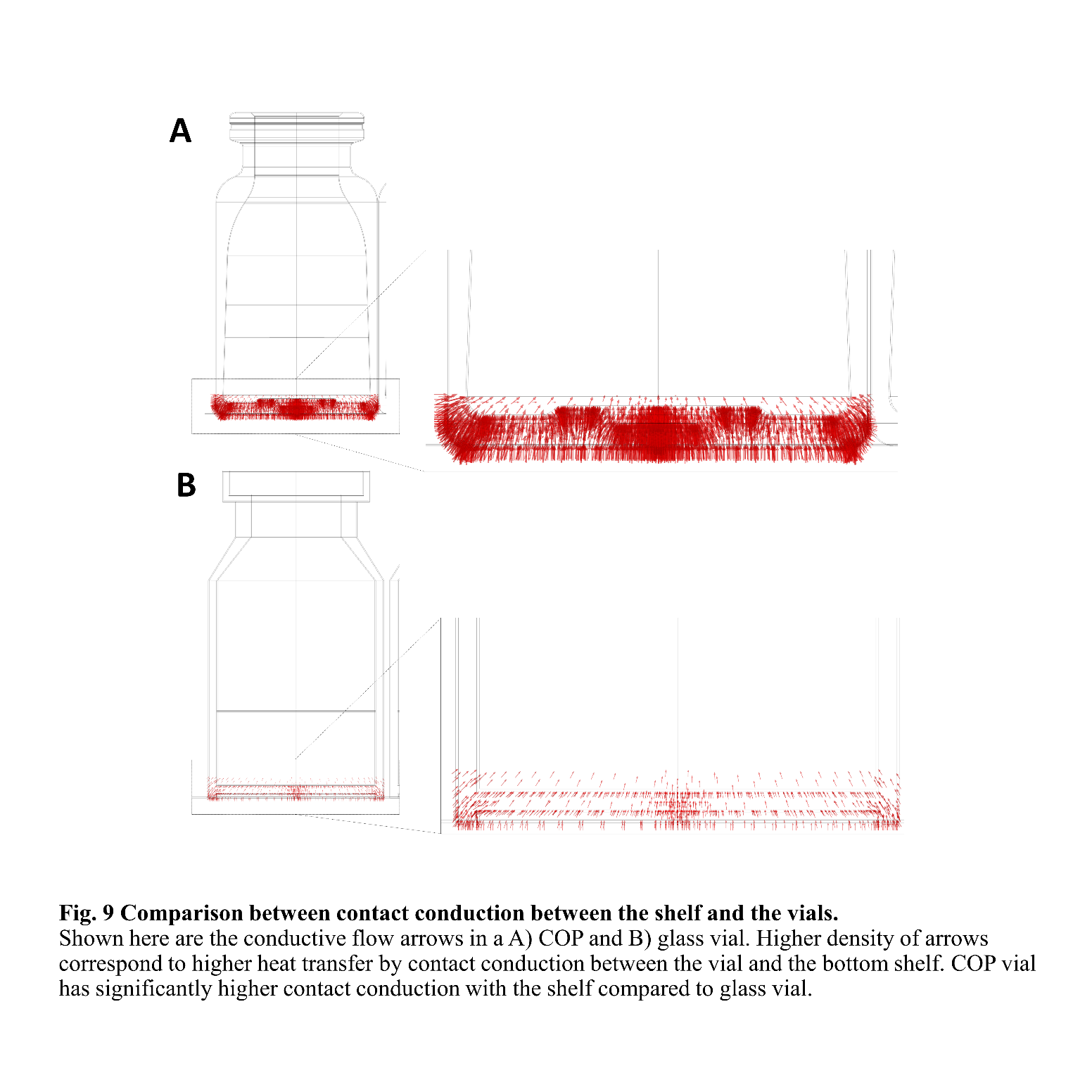


**Fig. S2 Comparison between contact conduction between the shelf and the vials.**Shown here are the conductive flow arrows in a A) hybrid COP and B) glass vial. Higher density of arrows corresponds to higher heat transfer by contact conduction between the vial and the bottom shelf. Hybrid COP vial has significantly higher contact conduction with the shelf compared to glass vial.


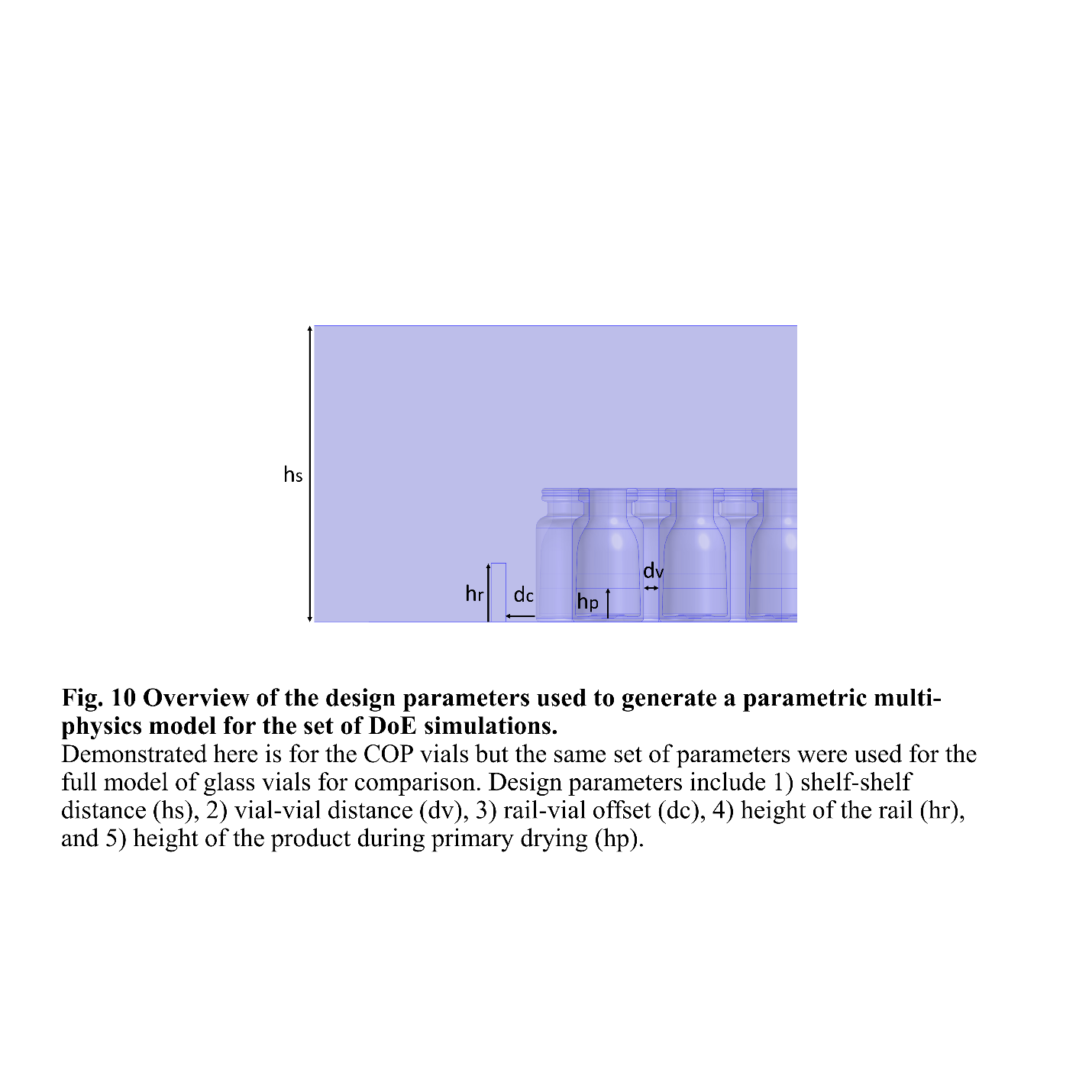


**Fig. S3 Overview of the design parameters used to generate a parametric multi-physics model for the set of DoE simulations.**Demonstrated here is for the hybrid COP vials but the same set of parameters were used for the full model of glass vials for comparison. Design parameters include 1) shelf-shelf distance (h_s_), 2) vial-vial distance (d_v_), 3) rail-vial offset (d_c_), 4) height of the rail (h_r_), and 5) height of the product during primary drying (h_p_).


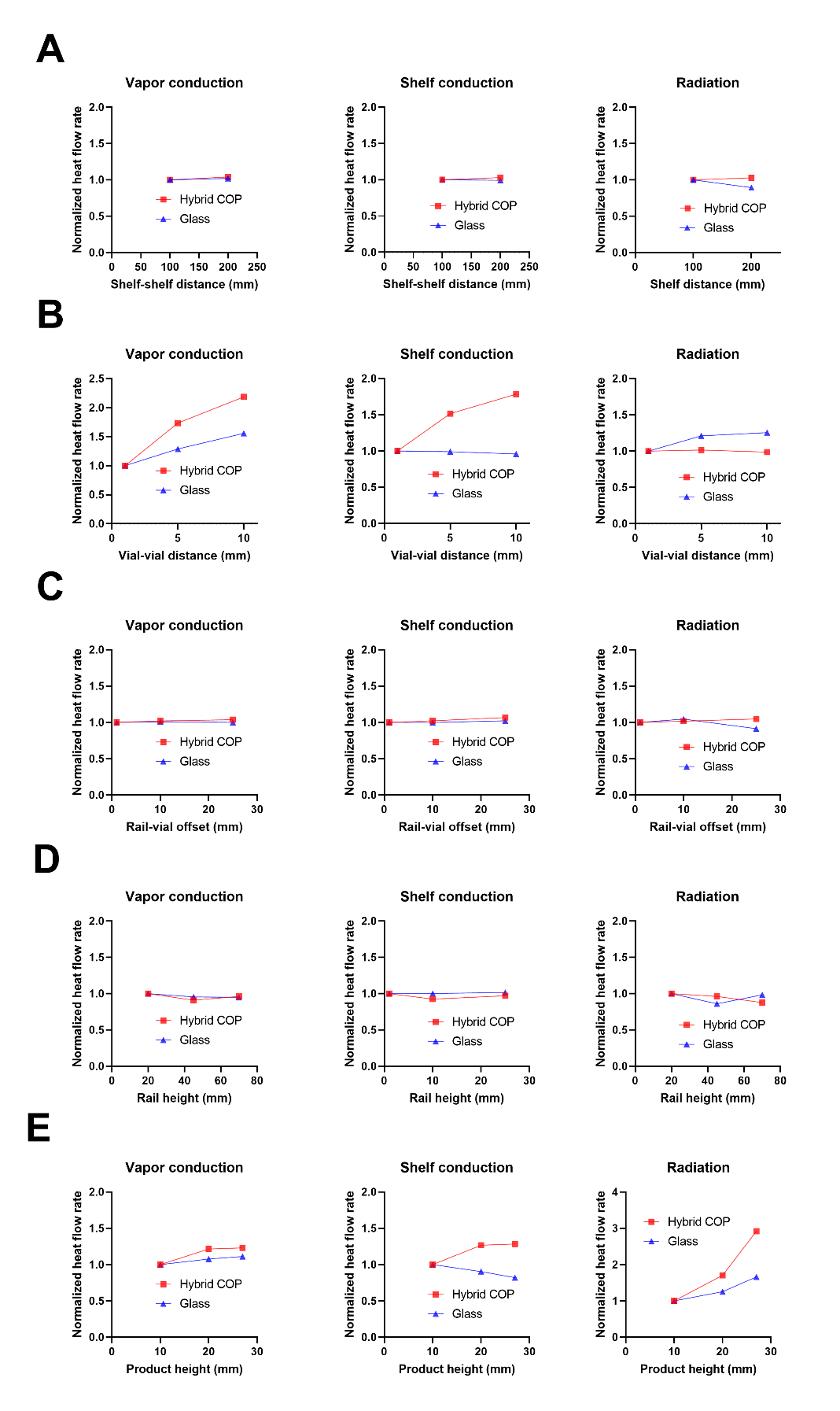


**Fig. S4. Understanding the impact of design parameters on each mode of heat transfer during primary drying, comparing hybrid COP vs glass vials.**Each column shows a specific mode of heat transfer and each row studies a certain design parameter. Datapoints show the normalized heat flow rate, averaged over all pressure values (30, 68, 112 mTorr) and all vials (C, E, and M). Design parameters include A) shelf-shelf distance, B) vial-vial distance, C) rail-vial offset, D) height of the rail, and E) height of the product during primary drying.

**
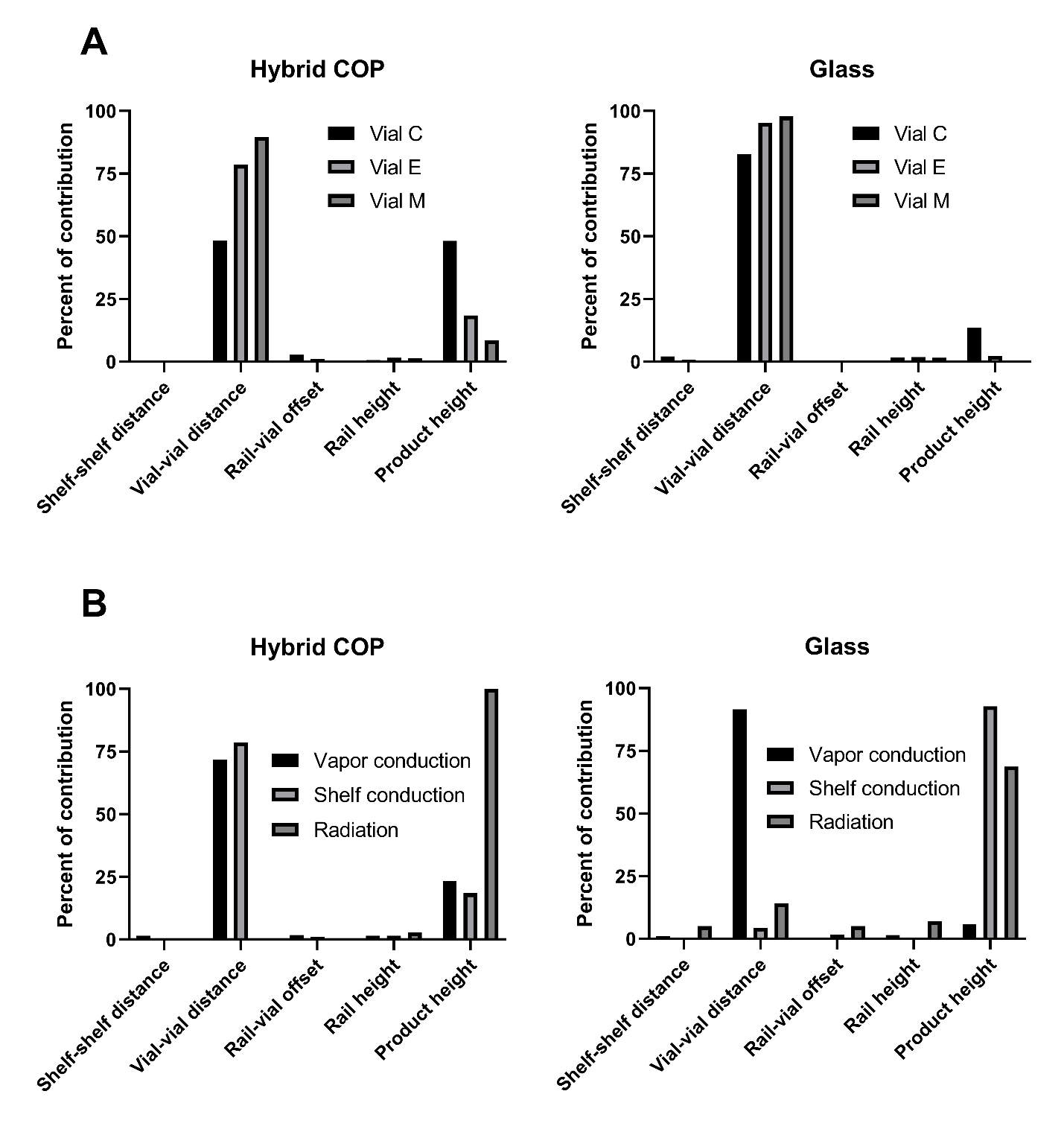
**

**Fig. S5. Results of ANOVA study to understand the importance of each design parameter in heat transfer during primary drying, comparing hybrid COP vs glass vials.**The percentage of contribution of each design parameter is demonstrated to A) the total heat flow rate or B) specific mode of heat transfer individually.
